# Probabilistic and machine-learning methods for predicting local rates of transcription elongation from nascent RNA sequencing data

**DOI:** 10.1093/nar/gkaf092

**Published:** 2025-02-18

**Authors:** Lingjie Liu, Yixin Zhao, Rebecca Hassett, Shushan Toneyan, Peter K Koo, Adam Siepel

**Affiliations:** Simons Center for Quantitative Biology, Cold Spring Harbor Laboratory, Cold Spring Harbor, NY 11724, United States; Graduate Program in Genetics, Stony Brook University, Stony Brook, NY 11794, United States; Simons Center for Quantitative Biology, Cold Spring Harbor Laboratory, Cold Spring Harbor, NY 11724, United States; Simons Center for Quantitative Biology, Cold Spring Harbor Laboratory, Cold Spring Harbor, NY 11724, United States; Simons Center for Quantitative Biology, Cold Spring Harbor Laboratory, Cold Spring Harbor, NY 11724, United States; Simons Center for Quantitative Biology, Cold Spring Harbor Laboratory, Cold Spring Harbor, NY 11724, United States; Simons Center for Quantitative Biology, Cold Spring Harbor Laboratory, Cold Spring Harbor, NY 11724, United States; Graduate Program in Genetics, Stony Brook University, Stony Brook, NY 11794, United States

## Abstract

Rates of transcription elongation vary within and across eukaryotic gene bodies. Here, we introduce new methods for predicting elongation rates from nascent RNA sequencing data. First, we devise a probabilistic model that predicts nucleotide-specific elongation rates as a generalized linear function of nearby genomic and epigenomic features. We validate this model with simulations and apply it to public PRO-seq (Precision Run-On Sequencing) and epigenomic data for four cell types, finding that reductions in local elongation rate are associated with cytosine nucleotides, DNA methylation, splice sites, RNA stem-loops, CTCF (CCCTC-binding factor) binding sites, and several histone marks, including H3K36me3 and H4K20me1. By contrast, increases in local elongation rate are associated with thymines, A+T-rich and low-complexity sequences, and H3K79me2 marks. We then introduce a convolutional neural network that improves our local rate predictions. Our analysis is the first to permit genome-wide predictions of relative nucleotide-specific elongation rates.

## Graphical Abstract

**Graphical Abstract:**
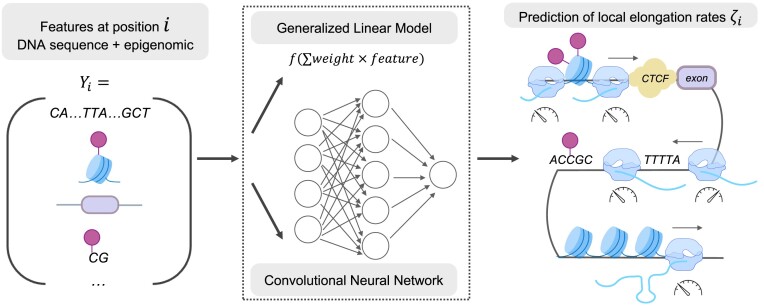

## Introduction

An enduring challenge in the study of eukaryotic gene regulation is that there is no single, well-defined point of control for gene expression. Instead, rates and patterns of expression are influenced at a broad array of cellular stages, ranging from pre-transcriptional chromatin remodeling to transcriptional, post-transcriptional, translational, and post-translational steps. Even within the critical stage of transcription—where research has traditionally focused on control of transcription initiation—many different steps can be regulated.

After RNA polymerase II (Pol II) has been recruited to a promoter, and together with its cofactors, unwound the DNA and established a stable RNA–DNA hybrid, it begins to translocate along the DNA template and synthesize a nascent RNA molecule [1, 2]. This process of productive elongation occurs at variable rates along the DNA template, sometimes pausing entirely for minutes at a time. A particular focus of recent research has been the tendency of Pol II to exhibit a pronounced pause ∼20–60-bp downstream of the TSS (Transcription Start Site). Such promoter-proximal pausing is remarkably widespread, both across metazoan species and across genes, and appears to be regulated in many cases [3, 4].

Rates of elongation also vary throughout the gene body, however, for reasons that are less well understood. What is known is, first, that productive elongation rates vary considerably across genes. In mammals, elongation through gene bodies occurs at an average rate of roughly 2 kb/min but this rate can vary by four-fold or more across genes, and it can also vary considerably for the same gene across cell types or conditions [5–10]. Second, the local elongation rate changes along each individual gene body, tending to increase with distance from the TSS but becoming reduced again at exons and near the termination site [5, 6]. Third, average elongation rates for genes are correlated with a wide variety of genomic and epigenomic features, including G+C content, exon density, nucleosome density, DNA methylation, histone marks such as H3K4me1, H3K4me3, H3K36me3, H3K79me2, and H4K20me1, stability of the DNA–RNA hybrid, the density of low-complexity sequences, as well as various DNA 5-mer frequencies [5–7, 9, 11, 12] (reviewed in [4, 13–15]). Fourth, elongation rates are positively correlated with the Pol II density itself, suggesting that the activity of one polymerase somehow facilitates the progress of others [5]. Finally, in at least some cases, elongation rates are actively regulated in response to various cellular stimuli, with dysregulation potentially contributing to disease progression [14].

Other studies have focused specifically on pausing of Pol II. Aside from the pronounced pausing that occurs proximal to the promoter, many, typically more subtle, pause sites occur within gene bodies, and, in the aggregate, these sites have a major effect on the dynamics of transcription elongation [16–19] (see also [20] for a recent study in yeast). These gene-body pause sites replicate well across experiments but vary substantially in their density across genes [18]. Such pausing has been reported to be associated with intron–exon boundaries, alternative splicing, certain properties of DNA shape and the RNA–DNA hybrid, DNA methylation, binding sites for factors such as ESR1, PAX5, SMAD3, YY1, and CTCF (CCCTC-binding factor), and particular sequence motifs, some of which are distinct from those associated with promoter-proximal pausing [18, 19, 21–23].

Despite these findings, much remains unclear about the determinants of local elongation rates through gene bodies. Most studies have either been based on the measurement of rates of progress of Pol II “waves” in time-course experiments [5–10], or on pre-identified gene-body pause sites [18, 19]. The wave experiments have limited genomic resolution, because waves tend to move tens of kilobases between timepoints, and are therefore better suited for evaluating correlates of average genic elongation rates than of local rates. They also are restricted to longer genes at which transcription can be induced or repressed. The experiments based on pre-identified pause sites have better genomic resolution but tend to reflect only the most extreme reductions in rate—ones sufficient to produce a statistically significant local peak in nascent RNA sequencing (NRS) read counts. Furthermore, with both types of studies, it is difficult to make sense of the observed genomic and epigenomic covariates because many of them are also strongly correlated with one another.

In this study, we revisit these questions using a fundamentally different statistical modeling approach. Our method is based on a recently developed “unified model” for NRS data, which describes both the kinetics of Pol II movement on the DNA template and the generation of NRS read counts reflecting the Pol II density along the genome (see bioRxiv: https://doi.org/10.1101/2021.01.12.426408) [24]. We adapt this model to allow for elongation rates that vary continuously along the genome, using a generalized linear model (GLM) to capture the relationship between the local rate and nearby genomic features. In this way, we avoid a dependency on predefined pause sites, and jointly consider the influences of many features on elongation rate. By accounting for differences across genes in initiation rate, we are able to efficiently pool information across genes, and extract high-resolution information about relative local elongation rates from steady-state data. This strategy avoids a dependency on specially designed time-course experiments, and enables application to a wide variety of existing public datasets. An extension to a convolutional neural network (CNN) enables nonlinear relationships to be captured and modestly improves prediction performance. We apply our methods to public data for four mammalian cell lines, identifying both previously known and novel correlates of elongation rate. We then use our models to predict nucleotide-specific elongation rates genome-wide and make our predictions available in a UCSC Genome Browser track.

## Materials and methods

### Unified probabilistic model for NRS data

**Figure 1.**
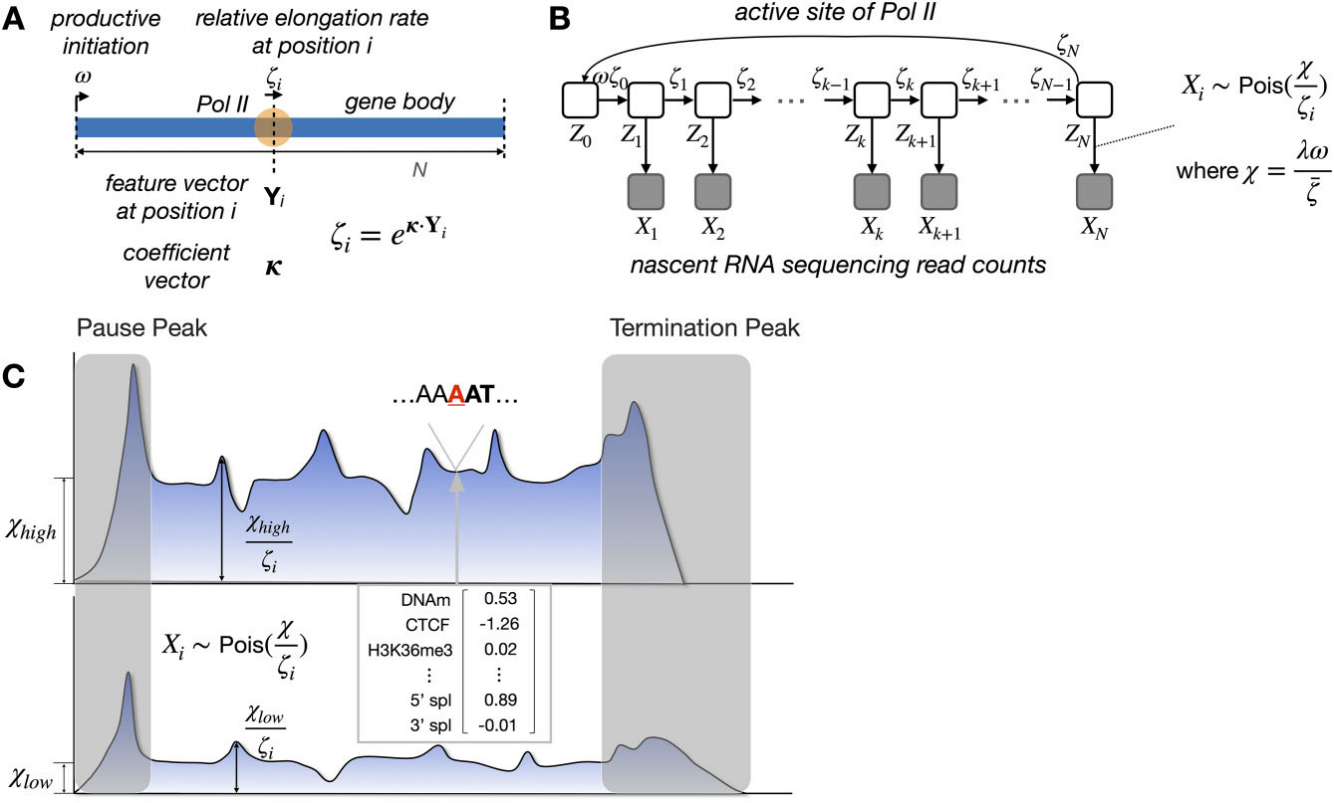
(**A**) Conceptual illustration of kinetic model for Pol II movement along DNA template in gene body. At nucleotide site *i*, relative elongation rate ζ_*i*_ is an exponentiated linear function of features $\boldsymbol{\mathbf {Y}}_i$ and coefficient κ. Promoter-proximal pausing and termination are ignored here. For simplicity, the subscript *j* for the gene is omitted here (see text). (**B**) Graphical model representation showing unobserved continuous-time Markov chain (*Z*_*i*_) and observed NRS read counts (*X*_*i*_). (**C**) Conceptual illustration showing that differences in average gene-body read depth are explained by the scaled initiation rate χ, while relative read depth is explained by the GLM for local elongation rate ζ_*i*_. Read count *X*_*i*_ is assumed to be Poisson-distributed with mean $\frac{\chi }{\zeta _i}$. Pause and termination peaks are omitted.

Our unified model has been detailed in [24]. Briefly, it consists of two layers: a continuous-time Markov model for the movement of individual polymerases along a transcription unit (TU), and a conditionally independent generating process for the read counts at each nucleotide site (Fig. 1). Together, these components produce a full generative model for NRS read counts along the TU, permitting inference of transcriptional rate parameters from the raw data. The model assumes (i) that collisions between polymerases are rare, allowing the movement of each one to be considered independently of the others and (ii) that premature termination of transcription is sufficiently rare that each polymerase can be assumed to traverse the entire DNA template if it is given enough time. (Notably, for this work, these assumptions only apply within gene bodies; they are unaffected, e.g. by premature termination upstream of or near the promoter-proximal pause site.)

The key extension for the purposes of this work is to allow for a different elongation rate at each nucleotide position, instead of a constant rate across all nucleotides. For reasons that will become clear below, we express the elongation rate for position *i* and gene *j* as a product of a gene-wide average elongation rate $\bar{\zeta }_j$ and a position-specific scale factor (hereafter, the “local elongation rate”), ζ_*i*, *j*_. In this version of the model, we ignore promoter-proximal pausing and termination and focus on the gene body, where the elongation signal is easiest to interpret. With these changes, the steady-state density for polymerase occupancy at nucleotide *i* along the body of gene *j* is given by


(1) \begin{eqnarray*} \pi _{i,j} = \frac{1}{\mathcal {Z}_j} \frac{\omega _j}{\bar{\zeta _j}\zeta _{i,j}}, \end{eqnarray*}

where ω_*j*_ is the gene-specific productive initiation rate and the normalization constant for gene *j* of length *N*_*j*_ is given by $\mathcal {Z}_j = \frac{\omega _j}{\bar{\zeta }_j} \sum _{i=1}^{N_j} \frac{1}{\zeta _{i,j}}$.

In turn, the local elongation rate ζ_*i*, *j*_ is defined by a generalized linear function of features along the genome:


(2) \begin{eqnarray*} \zeta _{i,j} = e^{\boldsymbol{\mathbf {\kappa }}\cdot \boldsymbol{\mathbf {Y}}_{i,j}}, \end{eqnarray*}

where $\boldsymbol{\mathbf {Y}}_{i,j}$ is the feature vector at site *i* of gene *j* and $\boldsymbol{\mathbf {\kappa }}$ is a corresponding vector of real-valued coefficients, whose first element is assumed to be a constant of 1 to accommodate an intercept for the linear function at the corresponding position in $\boldsymbol{\mathbf {\kappa }}$. The use of a single set of coefficients $\boldsymbol{\mathbf {\kappa }}$ for all analyzed sites allows sparse information about correlates of elongation rate to be pooled efficiently across many sites and many genes.

As in previous work [24], we assume that the NRS read counts *X*_*i*, *j*_ for nucleotide *i* of gene *j* are generated by a Poisson process, conditional on the steady-state density π_*i*, *j*_. In particular, we assume that $X_{i,j} \sim \text{Pois}\left(\frac{\lambda \omega _j}{\bar{\zeta _j}\zeta _{i,j}}\right)$, where λ is a scale parameter for sequencing read depth. Thus, the expected NRS read counts in gene *j* are proportional to the read depth and the productive initiation rate ω_*j*_ and inversely proportional to the gene-wide and local elongation rates $\bar{\zeta _j}$ and ζ_*i*, *j*_. The parameters λ, ω_*j*_, and $\bar{\zeta }_j$, however, are nonidentifiable from steady-state data; only the compound parameter $\chi _j = \frac{\lambda \omega _j}{\bar{\zeta }_j}$ can be estimated from the data. Thus, $X_{i,j} \sim \text{Pois}\left(\frac{\chi _j}{\zeta _{i,j}}\right)$.

With these assumptions, the joint log likelihood function for *M* independent genes is given by


(3) \begin{eqnarray*} \ell (\boldsymbol{\mathbf {X}}; \boldsymbol{\mathbf {\chi }}, \boldsymbol{\mathbf {\kappa }}, \boldsymbol{\mathbf {Y}}) &=& \sum _{j=1}^M \sum _{i=1}^{N_j} \log \left[ \frac{\left(\frac{\chi _j}{\zeta _{i,j}}\right)^{X_{i,j}}e^{-\frac{\chi _j}{\zeta _{i,j}}}}{X_{i,j}!} \right]\nonumber \\ &=& \sum _{j=1}^M \sum _{i=1}^{N_j} X_{i,j} \log \left( \frac{\chi _j}{\zeta _{i,j}} \right) -\frac{\chi _j}{\zeta _{j,i}} -\log \mathcal {Z} \nonumber \\ &=& \sum _{j=1}^M \sum _{i=1}^{N_j} X_{j,i} \log \left( \chi _j\right) - X_{j,i}\left(\boldsymbol{\mathbf {\kappa }}\cdot \boldsymbol{\mathbf {Y}}_{j,i}\right) -\chi _je^{-\boldsymbol{\mathbf {\kappa }}\cdot \boldsymbol{\mathbf {Y}}_{j,i}} - \log \mathcal {Z} \nonumber \\ &=& \sum _{j=1}^M s_{j}\log \left( \chi _j\right) - \boldsymbol{\mathbf {\kappa }}\cdot \boldsymbol{\mathbf {T}}_{j} - \chi _j U_{j} - \log \mathcal {Z}, \end{eqnarray*}

where $\mathcal {Z}$ does not depend on the free parameters and can be omitted in optimization, *s*_*j*_ is the sum of read counts for gene *j*, $s_j = \sum _{i=1}^{N_j}X_{i,j}$, and $\boldsymbol{\mathbf {T}}_{j}$ and *U*_*j*_ are defined analogously as


(4) \begin{eqnarray*} \boldsymbol{\mathbf {T}}_{j} &= \sum _{i=1}^{N_j} X_{i,j} \boldsymbol{\mathbf {Y}}_{i,j}, \end{eqnarray*}

(5) \begin{eqnarray*} U_{j} &= \sum _{i=1}^{N_j} e^{-\boldsymbol{\mathbf {\kappa }}\cdot \boldsymbol{\mathbf {Y}}_{i,j}}. \end{eqnarray*}

This joint log likelihood can be maximized easily by gradient ascent, in a manner similar to standard Poisson regression. The partial derivative with respect to the *n*th component of $\boldsymbol{\mathbf {\kappa }}$ is given by


(6) \begin{eqnarray*} \frac{\partial }{\partial \kappa _n} \ell (\boldsymbol{\mathbf {X}}; \boldsymbol{\mathbf {\chi }}, \boldsymbol{\mathbf {\kappa }}, \boldsymbol{\mathbf {Y}}) = \sum _{j=1}^M \chi _j\boldsymbol{\mathbf {V}}_{j,n} - \boldsymbol{\mathbf {T}}_{j,n}, \end{eqnarray*}

where the final subscript *n* indicates the *n*th element of a vector, and $\boldsymbol{\mathbf {V}}_{j}$ is defined as


(7) \begin{eqnarray*} \boldsymbol{\mathbf {V}}_{j} = \sum _{i=1}^{N_j} e^{-\boldsymbol{\mathbf {\kappa }}\cdot \boldsymbol{\mathbf {Y}}_{i,j}} \boldsymbol{\mathbf {Y}}_{i,j}. \end{eqnarray*}

For a given value of $\boldsymbol{\mathbf {\kappa }}$, the maximum for χ_*j*_ can be determined analytically as


(8) \begin{eqnarray*} \hat{\chi }_j = \frac{s_j}{\lambda U_j}, \end{eqnarray*}

where λ is approximated as the average read depth across all genes. Thus, the gradient ascent algorithm iteratively improves estimates of $\boldsymbol{\mathbf {\kappa }}$ and, on each iteration, fully optimizes χ_*j*_ conditional on the other parameters. Notice that the sufficient statistics *s*_*j*_ and $\boldsymbol{\mathbf {T}}_{j}$ need only be computed once, in preprocessing, but *U*_*j*_ and $\boldsymbol{\mathbf {V}}_{j}$ must be recomputed on each iteration of the gradient ascent algorithm. We used a learning rate of 10^−7^ (i.e. the multiplier for the gradient on each iteration) for gradient ascent.

### Penalized likelihood extension

In the case of a high-dimensional feature vector (i.e. with the *k*-mer or combined versions of the model), we augmented the log likelihood with a sparsity penalty. We experimented with L1 (lasso), L2 (ridge regression), and combined L1/L2 (elastic net) penalties but found the L1 version to work best in this setting. In this version, the objective function to maximize is (cf. equation 3)


(9) \begin{eqnarray*} \ell ^{\prime }(\boldsymbol{\mathbf {X}}; \boldsymbol{\mathbf {\chi }}, \boldsymbol{\mathbf {\kappa }}, \boldsymbol{\mathbf {Y}}, \nu ) = \sum _{j=1}^M \left[ s_{j}\log \left( \chi _j\right) - \boldsymbol{\mathbf {\kappa }}\cdot \boldsymbol{\mathbf {T}}_{j} - \chi _j U_{j}\right] - \nu \sum _n|\kappa _n|,\nonumber\\ \end{eqnarray*}

where ν is a hyperparameter determining the strength of the penalty and the final sum is over all features. Here, the partial derivative with respect to the *n*th component of $\boldsymbol{\mathbf {\kappa }}$ is given by


(10) \begin{eqnarray*} \frac{\partial }{\partial \kappa _n} \ell ^{\prime }(\boldsymbol{\mathbf {X}}; \boldsymbol{\mathbf {\chi }}, \boldsymbol{\mathbf {\kappa }}, \boldsymbol{\mathbf {Y}}, \nu ) = \sum _{j=1}^M \left[\chi _j\boldsymbol{\mathbf {V}}_{j,n} - \boldsymbol{\mathbf {T}}_{j,n}\right] - \nu \operatorname{sgn}(\kappa _n).\nonumber\\ \end{eqnarray*}

We determined a value for the hyperparameter ν separately for each analysis by cross-validation. Specifically, we set ν to the value that minimized the Akaike Information Criterion (AIC) for held-out testing data, using 80% of the data for training and 20% for testing (see Supplementary Fig. S8). We considered a grid of possible ν values, estimating $\boldsymbol{\mathbf {\kappa }}$ by maximizing the penalized log likelihood for the training data for each choice of ν, and then evaluating the AIC for the testing data. The estimate of $\boldsymbol{\mathbf {\kappa }}$ and ν that minimized the AIC was used for the subsequent analyses.

### DNA *k*-mer features

Two types of *k*-mer models were considered, including either 5-mers only or all *k*-mers for *k* ∈ {1, 2, 3, 4, 5}. In both cases, a separate indicator feature was defined for each candidate *k*-mer (1024 or 1364 features, respectively). At each site *i* and gene *j*, this feature was given a value of 1 if the reference genome matched that *k*-mer at the corresponding position and a value of 0 otherwise. For odd values of *k*, a match was evaluated by aligning the middle position of the *k*-mer with the reference base at position *i*, *j*, assuming that the middle position represented the active site of Pol II. For even values of *k*, position *k*/2 was aligned with the reference base, so that the active site fell slightly left of center in the *k*-mer, as shown by the underlined bases in Fig. 4. A separate set of experiments considered 5-mers shifted upstream or downstream of the active site (Fig. 5C and Supplementary Figs S18 and S19).

**Figure 2.**
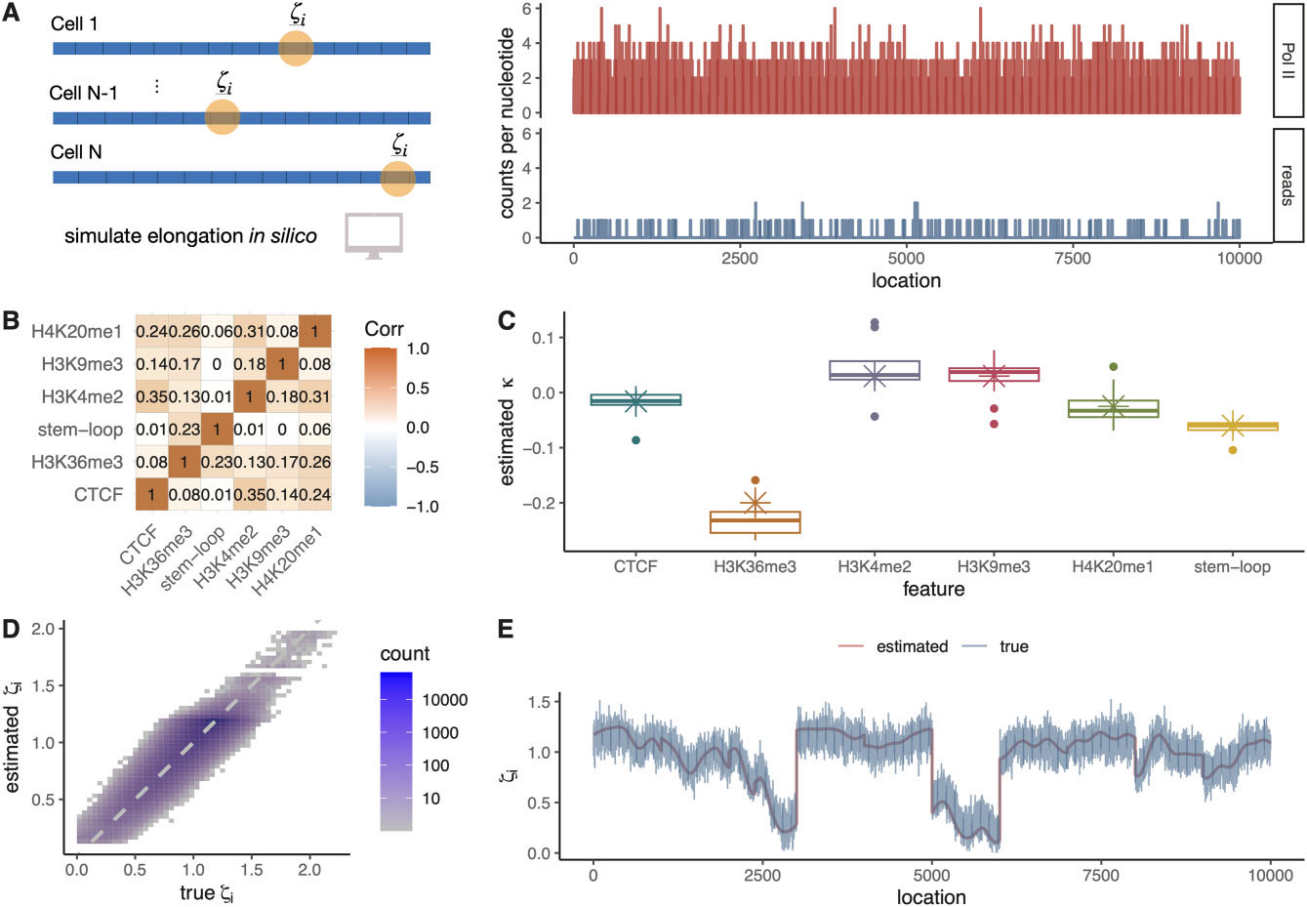
(**A**) The SimPol simulator tracks the movement of virtual polymerases across DNA templates in a population of cells (left panel). Once an equilibrium is reached, read counts per site are sampled in proportion to the simulated Pol II density, such that the average read depth is matched to real PRO-seq (Precision Run-On Sequencing) data (right panel). (**B**) Correlation map of selected epigenomic features for simulations (Spearman’s ρ). (**C**) Box plots for estimated coefficient κ in 10 replicates compared with ground truth in simulations (crosses). (**D**) Estimated versus true nucleotide-specific elongation rates ζ_*i*_ across all simulated TUs (*r*^2^ = 0.75). (**E**) Estimated versus true nucleotide-specific elongation rates ζ_*i*_ along an individual TU in 10 replicates (*r*^2^ = 0.87).

**Figure 3.**
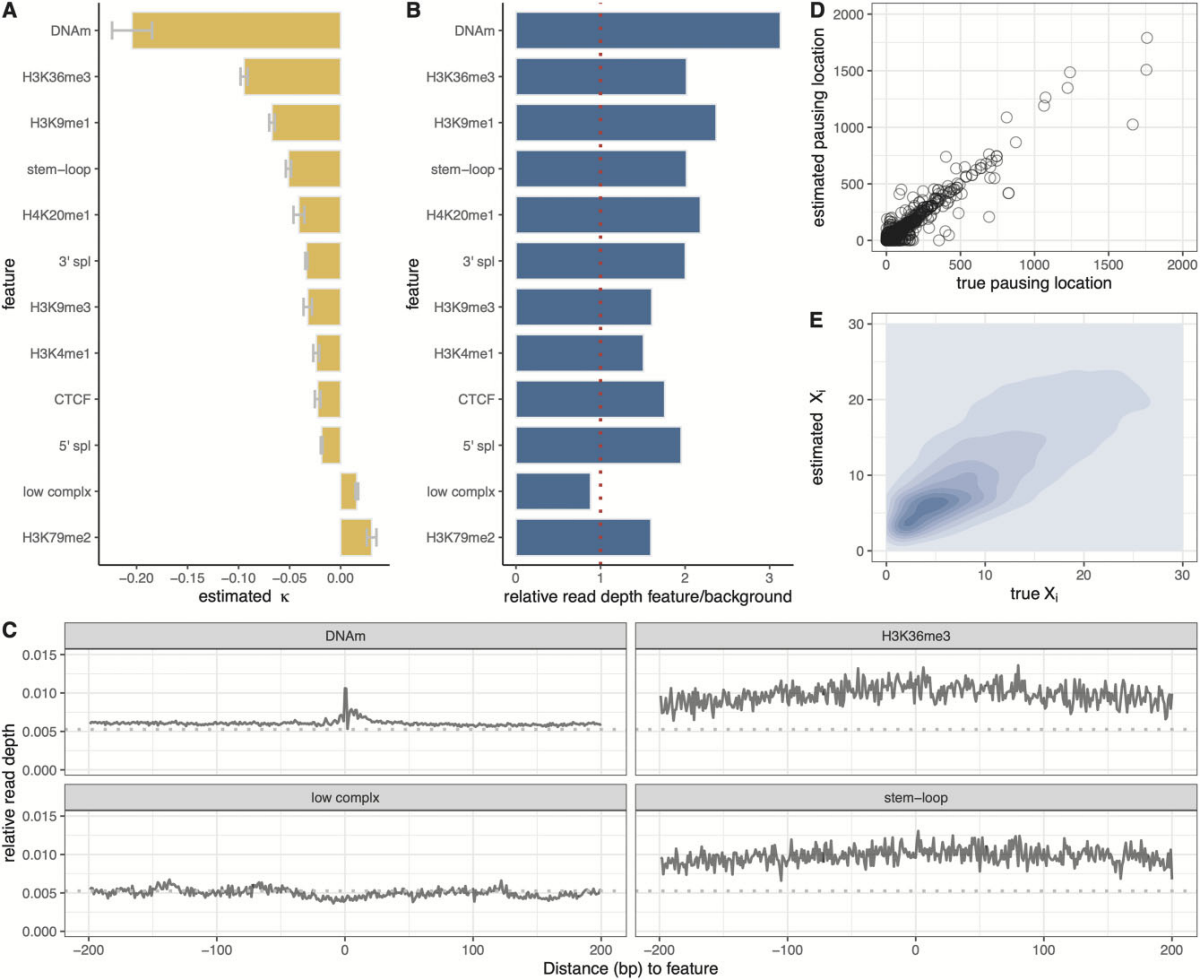
(**A**) Estimated coefficient κ for the 12 epigenomic features considered, based on PRO-seq data for K562 cells [25]. Sign indicates direction and absolute value indicates strength of association with local elongation rate. Error bars indicate one standard error in each direction. (**B**) Ratio of relative average PRO-seq read depth in regions covered by each feature to that in regions not covered by it (see text). (**C**) Metaplot (average values across sites) of relative read depths centered on four selected features. Dashed line represents average across all gene bodies. (**D)** Estimated versus true pausing locations within gene bodies (see text) (*r*^2^ = 0.68). (**E**) Predicted versus true PRO-seq read depths (*X*_*i*_) for held-out data averaged over 1-kb intervals for all TUs (*r*^2^ = 0.45).

**Figure 4.**
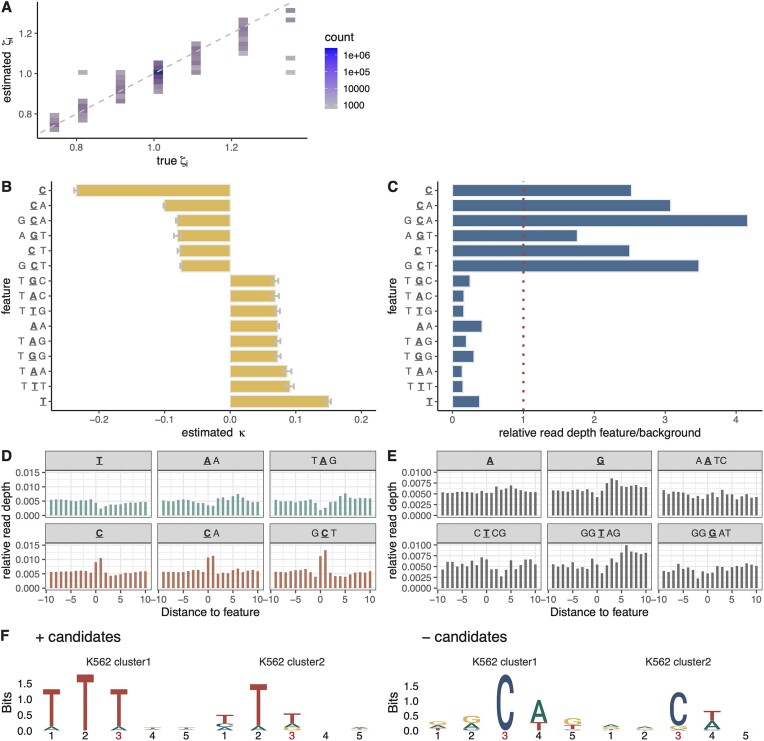
(**A**) Estimated versus true nucleotide-specific elongation rates ζ_*i*_ in 10 rounds of simulated *k*-mer data (*r*^2^ = 0.89). (**B**) Estimated coefficient κ for top *k*-mers (*k* ≤ 5) based on PRO-seq data for K562 cells [25]. Sign indicates direction and absolute value indicates strength of association with local elongation rate. The active site of Pol II is underlined. Error bars indicate one standard error in each direction. (**C**) Ratio of relative average PRO-seq read depth at sites associated with each *k*-mer to that at sites not associated with it (see text). (**D**) Metaplot of relative read depths for three *k*-mers with positive coefficients (top panel) and three with negative coefficients (bottom panel). (**E**) Metaplot of relative read depths for six *k*-mers having coefficients close to zero. (**F**) Sequence logos summarizing clusters of 5-mers five nucleotides centered on the active site that are positively (left panel) or negatively (right panel) associated with elongation rate.

**Figure 5.**
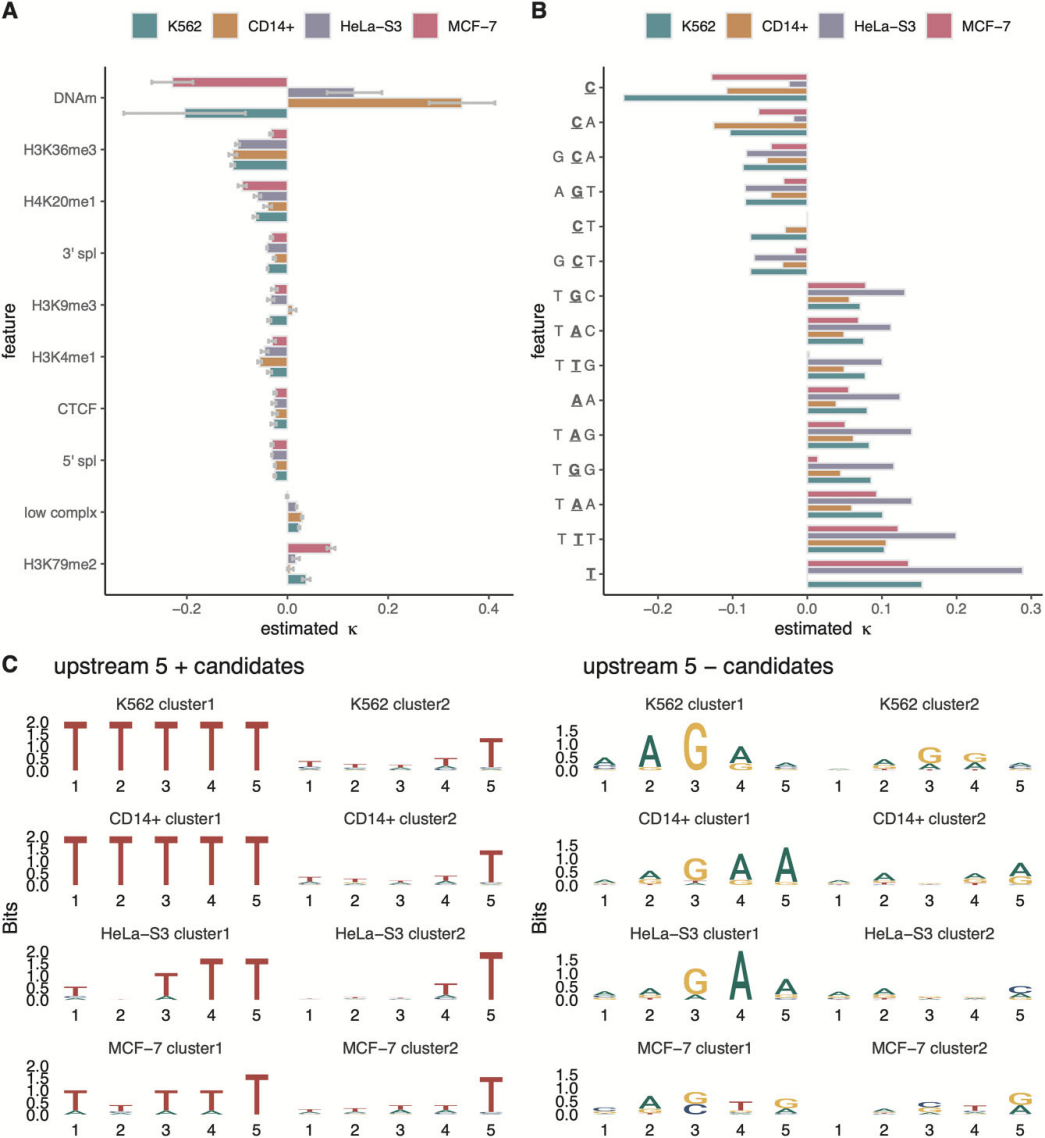
(**A**) Estimated coefficient κ for 10 epigenomic features based on PRO-seq data for four mammalian cell lines: K562 [25], CD14+ [26], HeLa-S3 [27], and MCF-7 [28]. Sign indicates direction and absolute value indicates strength of association with local elongation rate. Error bars indicate one standard error in each direction. (**B**) Estimated coefficients κ for top *k*-mers (*k* ≤ 5) in the same cell lines. The active site of Pol II is underlined. (**C**) Sequence logos summarizing clusters of 5-mers five nucleotides upstream of the active site that are positively (left panel) or negatively (right panel) associated with elongation rate (see “Materials and methods” section).

### Model extension allowing for sequence bias

We also implemented a version of the *k*-mer model that allows for some, potentially unknown, source of nucleotide bias at the 3′ ends of aligned PRO-seq reads, and attempts to find *k*-mer associations relative to that bias. The idea behind this model is to see if larger *k*-mer associations persist even if the nucleotides at the apparent active site are somehow biased by the protocol (see Supplementary Fig. S10C).

This version of the model allows for arbitrary relative frequencies of 3′ nucleotides π_A_, π_C_, π_G_, and π_T_, which in practice are pre-estimated from the bulk distribution of 3′ nucleotides in the PRO-seq reads. They are accommodated in the model by replacing the single read-depth scale parameter λ with a separate scale factor for each nucleotide, λ_A_, λ_C_, λ_G_, and λ_T_, such that for each base *b* ∈ {A, C, G, T}, λ_*b*_ = 4λπ_*b*_. For mathematical convenience, we then reparameterize using $\rho _b = \frac{\lambda _b}{\lambda } = 4\pi _b$.

After this generalization, the (unpenalized) log likelihood becomes (cf. equation 3)


(11) \begin{eqnarray*} && \ell (\boldsymbol{\mathbf {X}}; \boldsymbol{\mathbf {\chi }}, \boldsymbol{\mathbf {\kappa }}, \boldsymbol{\mathbf {Y}}) = \sum _{j=1}^M s_{j}\log \left( \chi _j\right) - \boldsymbol{\mathbf {\kappa }}\cdot \boldsymbol{\mathbf {T}}_{j}\nonumber\\ && \quad + \left[\sum _{i=1}^{N_j} X_{ij} \log \rho _i \right] - \chi _j U^{\prime }_{j} - \log \mathcal {Z}, \end{eqnarray*}

where we use the notation ρ_*i*_ to indicate the value of ρ_*b*_ corresponding to the nucleotide at position *i*, and where $U^{\prime }_j$ is like *U*_*j*_ (cf. equation 5) but has its terms weighted by the corresponding ρ_*b*_ parameters:


(12) \begin{eqnarray*} U^{\prime }_j &=& \sum _{i=1}^{N_j} \rho _i e^{-\boldsymbol{\mathbf {\kappa }}\cdot \boldsymbol{\mathbf {Y}}_{i,j}} \nonumber \\ &=& \sum _{i=1}^{N_j} e^{-(\boldsymbol{\mathbf {\kappa }}\cdot \boldsymbol{\mathbf {Y}}_{i,j} - \log \rho _i)}. \end{eqnarray*}

The effect of this model is to add to each dot product $\boldsymbol{\mathbf {\kappa }}\cdot \boldsymbol{\mathbf {Y}}_{i,j}$ a quantity of −log ρ_*i*_. Thus, nucleotides *b* that are overrepresented (with ρ_*b*_ > 1) are penalized, whereas nucleotides that are underrepresented (with ρ_*b*_ < 1) are rewarded. As a result, *k*-mer associations that simply reflect the background distribution are down-weighted and ones that represent departures from that distribution are up-weighted. If the nucleotides are uniformly distributed (with ρ_*b*_ = 1), the model collapses to the original version.

### Smoothing filters for genomic features

As specified, the model requires that any influence on the elongation rate at nucleotide *i* of gene *j* must be captured by the feature vector for the same position, $\boldsymbol{\mathbf {Y}}_{i,j}$. Some features, however, appear to have broader effects that spread out to adjoining nucleotide positions. For example, 3′ and 5′ splice sites are narrowly defined at a few nucleotide positions, but metaplots of NRS data suggest that their effects on elongation rate extend for as much as a hundred nucleotides (see e.g. Supplementary Fig. S6A–C), likely because physical interactions between Pol II and the spliceosome can occur over a fairly broad region.

To address this problem, we introduced a preprocessing device called a “smoothing filter” that can be applied to any genomic feature to cause its influence to be distributed to adjacent nucleotide positions. Even for features that are not narrowly defined at a few nucleotides, smoothing filters can be useful in compensating for different levels of genomic resolution across features, e.g. to put different ChIP-seq (Chromatin immunoprecipitation sequencing) datasets on the same genomic scale.

Formally, a filter *F*_*r*, σ, δ_ is a function defined by three parameters: a “radius” of application *r*, a “smoothing bandwidth” σ, and an “offset” δ. Applying a filter requires replacing each (scalar) covariate $Y_{i,j}^{(n)}$ with a filtered version, $\bar{Y}_{i,j}^{(n)}$, such that


(13) \begin{eqnarray*} \bar{Y}_{i,j}^{(n)} = \frac{1}{\mathcal {Z}} \sum _{k=-r}^{+r} Y_{i+k+\delta ,j}^{(n)} F_{r,\sigma ,\delta }(k),\qquad \text{where } \mathcal {Z} = \sum _{k=-r}^{+r} F_{r,\sigma ,\delta }(k).\nonumber\\ \end{eqnarray*}

The filter *F*_*r*, σ, δ_(*k*) can take a variety of functional forms, and simple filters can be composed to create more complex ones. In this work, however, we found it most useful to work with a Gaussian filter,


(14) \begin{eqnarray*} F_{r,\sigma ,\delta }(k) = \frac{1}{\sigma }e^{-\frac{1}{2} \left( k/\sigma \right)^2}, \end{eqnarray*}

and a generalized filter,


(15) \begin{eqnarray*} F_{r,\sigma ,\delta }(k) = \lambda _{k+r}, \end{eqnarray*}

which is defined by a vector of nonnegative scale factors $\boldsymbol{\mathbf {\lambda }}= \lbrace \lambda _0, ..., \lambda _{2r}\rbrace$ that were estimated from metaplots of PRO-seq data centered on the feature of interest. We used the generalized filter for the 3′ and 5′ splice-site features, and the Gaussian filter for the ChIP-seq-based features, including the histone modifications and CTCF (*r* = 400 bp, σ = 100 bp; see examples in Supplementary Fig. S6). We also applied a Gaussian filter (with *r* = 500 bp, σ = 200 bp) to the stem-loop feature, matching it to the corresponding metaplot.

### Standardization of feature values

As with any linear modeling application, the epigenomic and *k*-mer features needed to be standardized to allow the estimated coefficients to be on the same scale and comparable with one another. We used the simple approach of shifting and rescaling the values for each feature to have a mean of zero and standard deviation of one. Some features were defined for only a subset of genomic positions (such as DNA methylation, which was relevant only at CpG dinucleotides); in these cases, the defined values were first standardized and the remaining positions were subsequently assigned values of zero, to ensure that they had no positive or negative effect in the linear model. Notably, in the case of DNA methylation, this strategy ensures that the model can distinguish between the effects of methylation and those from cytosine nucleotides, because no signal comes from noncytosine positions.

Standardization of the indicator features for *k*-mers led to a computational problem that required special attention. Prior to standardization, these features had values of zero at the vast majority of genomic positions, but after standardization these zeroes were converted to negative real values. As a result, the calculation of the *U*_*j*_ and $\boldsymbol{\mathbf {V}}_j$ values needed for each iteration of the gradient ascent algorithm (equations 5 and 7) became considerably more laborious. We addressed this problem by first calculating the *U*_*j*_ and $\boldsymbol{\mathbf {V}}_j$ values from the unstandardized feature values (containing mostly zeroes) and using a linear transformation to convert them to the corresponding values for the standardized features. In this way, the speed of processing the unstandardized values could be maintained while properly considering the effects of standardization.

### SimPol simulator

The SimPol (Simulator of Polymerases) program tracks the independent movement of RNA polymerases (RNAPs) along the DNA templates of a large number of cells (Fig. 2A). As detailed in [24], the original program accepts user-specified parameters for the initiation pause-escape, and elongation rates, as well as the number of cells being simulated, the gene length, and the total time of transcription. For this study, we modified the simulator to accept a vector of position-specific elongation rates, which could be pre-computed based on covariates (see below). We also omitted the pause-escape portion of the simulation model. Based on the specified parameters, the simulator simply allows each polymerase to move forward or not at the specified rates, in time slices of 10^−4^ min, assuming at most one movement per time slice, and prohibiting movement if another polymerase blocks forward progress. The program was run until polymerase occupancy along the gene body reached equilibrium (20 min simulated time), and then the empirical density was output as a file in csv format. From this output, an accompanying R script was used to sample synthetic NRS read counts at each nucleotide position (as in [24]), with a target mean read depth equal to that observed in the real PRO-seq data for K562 cells (Supplementary Fig. S1B).

### Generation of synthetic NRS data

For the epigenomic simulations, we used SimPol to simulate 10 replicates of 100 TUs of length 10 kb in 5000 cells. In each replicate, gene-specific initiation rates were sampled from real data for K562 cells [24], by rescaling the estimated χ values as initiation rates α having a median of 1 event per min. Of the 100 simulated TUs in each replicate, 20 were held out for testing, as noted in the text. We selected six representative epigenomic features from real data for K562 cells: CTCF binding sites, four histone marks, and RNA stem-loops (see Fig. 2B). To generate synthetic covariates, we sampled combinations of these covariates from the real data in 1 kb blocks (Supplementary Fig. S1C and D), ensuring that their correlation structure was preserved. We then generated feature-specific local elongation rates according to the same GLM used for inference, but with the addition of Gaussian noise. Specifically, we assigned a κ coefficient to each feature and set it equal to a value estimated in a preliminary analysis of the K562 data, and then we set the local elongation rate for each position *i* to be $\zeta _i = \exp (\boldsymbol{\mathbf {\kappa }}\cdot \boldsymbol{\mathbf {Y}}_i) + \delta _i$, where $\delta _i \sim \mathcal {N}(0,0.1)$. The vector of simulated ζ_*i*_ values was then passed to SimPol for simulation of polymerase movement and NRS read counts (above). We also experimented with a version of these simulations without Gaussian noise (Supplementary Fig. S3A–D).

The simulations for the 5-mer model were similar, but in this case we simulated 200 TUs in each replicate, owing to the high-dimensional feature vector. We randomly sampled 100 5-mers and assigned them coefficients ranging from −0.3 to 0.3, while the remaining 5-mers were assigned a coefficient of 0. The initiation rates and position-specific elongation rates were determined as above, but without the addition of Gaussian noise.

### Analysis of real data

We acquired PRO-seq datasets for K562, CD14+, Hela-S3, and MCF-7 cell lines from published sources [25–28] and processed them using the proseq2.0 pipeline (https://github.com/Danko-Lab/proseq2.0) [29]. The data were processed exactly as described in [24]. Briefly, mapping was performed with human genome assembly GRCh38.p13. The 3′ ends of mapped reads—which we take to represent the active sites of transcriptionally engaged polymerases—were recorded in bigWig files and used for analysis. Gene annotations were downloaded from Ensembl (release 99) in GTF [30]. Annotations of protein-coding genes from the autosomes and sex chromosomes were used, excluding overlapping genes on the same strand. DENR (Deconvolution of Expression for Nascent RNA-sequencing data) [26] was applied separately to each dataset to select dominant pre-messenger RNA (pre-mRNA) isoforms and estimate corresponding expression levels. Genes with a DENR-estimated abundance of <10 TPM (transcripts per kilobase million) were removed.

We took several measures to refine gene annotations, select regions for analysis, and remove signals not representative of elongation rates. First, we refined the annotated TSS positions using cell-type-matched NRS data. For the K562 data, we used available CoPRO-cap (Coordinated Precision Run-On and sequencing with 5′ capped RNA) data [31] to re-position the TSS within a region −1500 bp to +1500 bp of the annotated TSS selected by DENR. In the other three cell lines, we performed a similar refinement using the 5′ ends of aligned PRO-seq reads (as in [24]). Second, we conservatively defined the “gene body” for each gene as the interval from 2250-bp downstream of the refined TSS (a distance we verified was sufficient to eliminate all pause peaks) to 250-bp upstream of the annotated TTS. (See Supplementary Fig. S25 for a comparison with a more aggressive filtering strategy, which has almost no impact on our results.) Gene bodies <6 kb were omitted. Finally, to eliminate potential internal TSSs—which create PRO-seq peaks not representative of gene-body elongation rates—we used GRO-cap (Global Nuclear Run-On and sequencing with 5′ capped RNA) [32] or PRO-cap (Precision Run-On and sequencing with 5′ capped RNA) [33] data where available, as well as dREG (discriminative Regulatory Element detection from GRO-seq) [34] predictions from the primary PRO-seq data. Specifically, we masked out predicted dREG peaks and 2-kb intervals centered on GRO-cap or PRO-cap peaks (with read counts >10) (see example in Supplementary Fig. S7). In later analyses (with MCF-7), we omitted the less reliable dREG filter and used PRO-cap only. The final datasets consisted of 6391 genes for K562, 5336 for CD14+, 6657 for HeLa, and 6193 for MCF-7. For the comparison of cell lines, we considered the intersection of these sets, which consisted of 3716 genes.

To eliminate the shared “U-shaped” pattern along gene bodies, we first merged the data for all genes after standardizing their lengths to a common scale and normalizing the raw read counts by dividing them by the median for each gene (see Supplementary Fig. S26). We then smoothed the merged data using the LOESS method, creating a gently U-shaped curve with an average height of one. We then adjusted the raw read counts by dividing by the height of the LOESS curve at the corresponding positions, ensuring that the data were flat on average. These adjusted read counts served as the inputs *X*_*i*, *j*_ for our model.

For the nonmodel-based validation of relative read depths, we first computed a relative read depth along each gene body by dividing all read counts by the average value within each gene body, to account for differences in initiation and/or average elongation rates. We then computed the ratio of the relative read depth within the “covered” regions to that in “uncovered” regions, pooling data across all 6000 robustly expressed genes. A ratio of >1 therefore indicates an increase in PRO-seq read depth at annotated sites, whereas a ratio of <1 indicates a decrease in read depth.

For the analysis of pausing locations within gene bodies (Fig. 3E and Supplementary Fig. S10A), we partitioned each gene body into 200-bp intervals, summed the PRO-seq read counts within each window, and identified the top five intervals for each gene as putative pausing locations.

### Detailed analysis of differences across cell types

Initially we found a striking difference across cell types in the apparent association of DNA methylation with elongation rate, which was strongly negative in K562 and MCF-7 cells, but strongly positive in CD14+ and HeLa-S3 cells (Fig. 5A). Further investigation showed, however, that this difference could be explained by a failure to efficiently filter out internal TSSs in CD14+ and HeLa-S3 cells, owing to the lack of GRO-cap or PRO-cap data for these cell types. An analysis of K562 cells with and without the filter for internal TSSs showed clearly that the correlation with DNA methylation is highly sensitive to the filter (Supplementary Fig. S12). Because these internal TSSs tend to be both unmethylated and transcribed, leading to a high PRO-seq read depth, they appear in the context of our model to suggest that unmethylated DNA has a reduced elongation rate, implying a positive correlation between methylation and elongation rate. This spurious signal of positive correlation overwhelms the true signal for negative correlation that occurs elsewhere throughout the gene bodies. We therefore disregard the positive κ estimates for CD14+ and HeLa-S3 cells as artifacts of unfiltered internal TSSs.

We also found that the positive association of H3K79me2 with local elongation rate was considerably strengthened in MCF-7 cells, whereas the positive association of low-complexity sequences was lost in this cell type. These differences traced back to an anomalous pattern of relative read depths around these two features in the MCF-7 data, with increases in H3K79me2 and decreases in low-complexity sequences relative to the background, for reasons we could not discern. Conversely, the negative coefficient for H3K36me3 was smaller in MCF-7 cells. These differences contributed to the reduced global correlation between MCF-7 and the other cell lines—particularly with CD14+, which itself was an outlier in having a positive H3K9me3 coefficient.

Differences across cell types were also observed in the strength of association of cytosines with elongation rate (Supplementary Fig. S15B). In particular, this association was largely absent in HeLa-S3 cells. On further investigation, however, we found that the HeLa-S3 dataset had an anomalous bulk distribution of 3′ nucleotides, with much less enrichment for cytosines than the others (Supplementary Fig. S27). This difference may also help to explain the unusually strong positive correlation for T-containing *k*-mers in HeLa-S3 cells. Despite reprocessing the raw data and re-mapping the reads for HeLa-S3, we were unable to uncover the reasons for this difference. Notably, the CD14+ dataset is based on an unequally concentrated four-dNTP library, where CTP and UTP concentrations are 10 times larger than ATP and GTP. Nevertheless, the bulk distribution of 3′ bases was similar to those for the other two-dNTP libraries.

### Test for ligation bias

To test for a nucleotide bias in ligation, we leveraged the particular adapter design of another CD14+ PRO-seq library, as follows. This library differs from the CD14+ library used in the main analysis as it is a one-dNTP (CTP) run-on library, which is not suitable for the application of our model. However, it can still serve as a useful dataset for studying ligation bias. These adapter designs, denoted as RA3 and RA5, incorporate a UMI (Unique Molecular Identifiers) consisting of random bases (NNNNNN) at the 3′ end of the 5′ adapter (see Supplementary Fig. S11C). In this configuration, the 3′ end of a UMI mirrors the 3′ end of an insert, allowing for a natural comparison of the nucleotide composition of insert–adapter dimers with no-insert adapter dimers. We first established that the 3′ ends of the synthesized UMIs have a uniform distribution of nucleotides (Supplementary Fig. S11D). Next, we examined the 3′ ends of UMIs that were either ligated to RA3 (no inserts) or inserts (with inserts). In a pool of ∼20 million sequenced reads, about ∼90% were insert–adaptor dimers, and ∼3% were no-insert adapter dimers. We found no major difference between the 3′-most nucleotides in these two sets (Supplementary Fig. S11E), suggesting no ligation bias. In particular, the cytosine frequencies in the two sets were nearly identical.

### Enrichment logos for 5-mer candidates

To determine the enrichment logos of the 5-mer model, we clustered them using a *K*-means algorithm as implemented in the *kmeans()* function in R (Hartigan–Wong algorithm). The 5-mers were divided into two groups based on their positive or negative κ values, with the top 50 candidates selected for each group. For each group, 5-mers with similar κ values were clustered with *K* = 2. The enrichment of the sequence logos for each cluster was visualized using *ggseqlogo* [35].

### Convolutional neural network

The CNN consists of separate branches for the epigenomic and sequence features (Supplementary Fig. S22). Input data were divided into subsequences of 100 bp with batch sizes of 64, leading to input dimensions of (64, 100, 10) for the epigenomic features and (64, 100, 4) for one-hot encoded DNA sequences. The epigenomic data branch consists of a single convolutional layer, whereas the sequence data branch uses a sequence of three convolutional layers of increasing channel and kernel sizes (as shown in Supplementary Fig. S22) to capture features and motifs of varying sizes. We used odd-sized kernels and the padding=“same” setting for the convolutional layers to preserve data dimensionality. Each convolutional layer is followed by a ReLU activation layer to allow for nonlinearity and a dropout layer set to 0.5 to prevent overfitting. The final layers concatenate and integrate features from the two convolutional branches while preserving data dimensionality. We experimented with several variants of this model architecture and found this one to perform best.

To match the CNN to our Poisson-based GLM, the output of the CNN at each nucleotide is assumed to represent ρ_*i*, *j*_ = log (ζ_*i*, *j*_), where ζ_*i*, *j*_ is the local elongation rate. The loss with respect to PRO-seq read counts *X*_*i*, *j*_ is then calculated as


(16) \begin{eqnarray*} \ell = X_{i,j}\rho _{i,j} + \chi _je^{-\rho _{i,j}}, \end{eqnarray*}

where χ_*j*_ is pre-estimated under the assumptions of the GLM and passed in to the model. This loss function is equivalent to the negative log likelihood under the Poisson model up to a constant that does not depend on the CNN parameters.

The data were split into a training set of ∼2.5k genes and a validation set of ∼300 genes. The model was trained with subsequences of length 100 but validation was performed on the full-length sequences. A dynamic learning rate scheduler was used to decrease the learning rate by half during training whenever the validation loss increased. Early stopping was used to optimize model performance and prevent overfitting. This entailed terminating training when the validation loss failed to decrease significantly over five consecutive epochs, usually after 20–30 epochs. Hyperparameter sweeping was used for determining optimal channel sizes and kernel sizes in each convolutional branch as well as for the learning rate and dropout.

## Results

### A GLM for variable elongation rates

Our previous unified model describes both the stochastic movement of Pol II along a DNA template and the generation of NRS read counts from underlying Pol II densities. The model has two layers: the first layer describes the movement of Pol II using the framework of continuous-time Markov chains [36]; and the second layer describes the generation of read counts at each site by assuming Poisson sampling conditional on Pol II density. We recently adapted this model to characterize the equilibrium dynamics of transcription initiation and promoter-proximal pausing [24], ignoring variability in elongation rate throughout the gene body. Here, we take a complementary approach, focusing on gene-body elongation rates but ignoring promoter-proximal pausing. We focus in particular on the relationships between local elongation rates and various genomic and epigenomic features (Fig. 1).

A key challenge in this analysis is that the information about elongation rate at each site is provided only by local changes in NRS read depth, which are difficult to detect reliably in low-coverage sequence data. We address this problem by describing the relationship between the genomic features at each site *i* in gene *j*, denoted $\boldsymbol{\mathbf {Y}}_{i,j}$, and the elongation rate at that site, ζ_*i*, *j*_, by a generalized linear relationship, $\zeta _{i,j} = \exp (\boldsymbol{\mathbf {\kappa }}\cdot \boldsymbol{\mathbf {Y}}_{i,j})$, where $\boldsymbol{\mathbf {\kappa }}$ is a vector of coefficients that is shared across all sites and all genes (Fig. 1A and B). Thus, as the model is fitted to the data, it “learns” to predict the site-specific elongation rate from local features through a simple linear function, which is exponentiated to ensure that the predicted rate remains nonnegative. In this way, the model can efficiently pool information about local rates across many sites in the genome and circumvent the problem of noise at each site.

A second challenge is that NRS read counts reflect differences in gene-level initiation rates as well as both gene-level and local elongation rates (Fig. 1C). In the same way that the density of cars along a highway reflects both the rate at which new cars enter the road and their average speed, the polymerase density along each gene body reflects both the productive initiation rate and the average elongation rate. In particular, the steady-state read depth within each gene body *j* can be shown to be proportional to the ratio of the productive initiation rate ω_*j*_ to the average elongation rate $\bar{\zeta }_j$ [24], making these two influences indistinguishable from steady-state data. Therefore, we focus instead on “local” variability in elongation rate. First, we estimate a separate compound parameter χ_*j*_ for each gene *j*, which can be interpreted as a read-depth-scaled initiation-to-elongation rate ratio for the gene as a whole ($\chi _j=\frac{\lambda \omega _j}{\bar{\zeta }_j}$; see “Materials and methods” section). Then we allow each site *i* in gene *j* to have a different “relative” local elongation rate ζ_*i*, *j*_, which is defined as a function of the local features via the GLM. These ζ_*i*, *j*_ values can be thought of as scale factors for the average elongation rate, taking values <1 for local slow-downs and values >1 for local speed-ups in polymerase movement. The model accounts for the observed read depth *X*_*i*, *j*_ at each site *i* by assuming *X*_*i*, *j*_ is Poisson-distributed with mean χ_*i*_/ζ_*i*, *j*_.

### The model recovers true elongation rates and epigenetic correlates in realistic simulations

We first tested our modeling approach on data simulated with SimPol [24], which tracks the movement of individual RNAPs along the DNA templates in thousands of cells under user-defined rates, and then samples NRS read counts in proportion to the steady-state polymerase density (Fig. 2A; see “Materials and methods” section). For this study, we extended SimPol to consider synthetic epigenomic correlates, which we tiled along each synthetic DNA template using a block-sampling approach based on real data for CTCF transcription binding sites, four different histone marks, and RNA stem-loops (Fig. 2B and Supplementary Fig. S1; see “Materials and methods” section). The “true” elongation rate at each site was determined by an exponentiated linear function of the associated covariates plus independent Gaussian noise. Overall, the estimated coefficients showed excellent agreement with the true values, with some variation across replicates (Fig. 2C). We did observe a slight estimation bias in some cases (e.g. H3K36me3), perhaps owing to unmodeled correlations between covariates. In most cases, however, the estimated coefficients were approximately unbiased, with median values close to the truth. A second experiment based on overdispersed sampling of the coefficients showed that the performance of the model remains excellent even when the true values are fairly far from those observed in real data (Supplementary Fig. S2).

In a third experiment, we estimated the coefficient vector $\boldsymbol{\mathbf {\kappa }}$ from training data and then predicted per-nucleotide values of the elongation rate ζ_*i*_ in held-out testing data (see “Materials and methods” section). In this setting, where the “true” values also reflect a GLM, the predicted elongation rates were well correlated with the true values (*r*^2^ = 0.75; Fig. 2D). A version without the addition of Gaussian noise showed almost perfect performance (Supplementary Fig. S3A–D). When visualized along an individual TU, the predictions form a smooth line through a cloud of variable true rates (Fig. 2E). While the precise degree of predictivity in these experiments depends on the details of our simulation scheme, these results nevertheless demonstrate that our model can accurately predict values of ζ_*i*_ when informative covariates are available.

### Several epigenomic and sequence features are correlated with local elongation rate

Having established that our model works well with simulated data, we applied it to real PRO-seq data from K562 cells [25], for which abundant epigenomic data are available. Based on previous reports [5–8], we selected 12 features as covariates: 5′ and 3′ splice sites evident from cell-type-matched RNA-seq data, DNA methylation based on whole-genome bisulfite sequencing data, CTCF binding sites based on ChIP-seq data, six histone modifications (H3K4me1, H3K9me1, H3K9me3, H3K36me3, H4K20me1, and H3K79me2) based on ChIP-seq data (all from ENCODE [37]), as well as apparent RNA stem-loops based on dimethyl sulfate data [38] and low-complexity sequences annotated in the UCSC Genome Browser [39]. At this stage, we omitted features strongly correlated with the DNA-sequence base composition, such as DNA melting temperature and stability of the DNA–RNA duplex. We also excluded several histone marks whose elongation-rate association is driven by the 5′ ends of TUs (see Supplementary Fig. S4) and we avoided explicit consideration of exon annotations, which are indirectly captured by splice sites and exon-associated H3K36me3 marks (see Supplementary Fig. S5). To account for differences in resolution or precision among these features, we devised smoothing filters for several of them, to distribute information along the genome sequence in an appropriate manner (see “Materials and methods” section and Supplementary Fig. S6).

Before analyzing the PRO-seq data, we carefully preprocessed them to avoid confounding signals not representative of local elongation rates (see “Materials and methods” section for details). Briefly, we adjusted annotated TSSs based either on cell-type-matched CoPRO-cap data [31], if available, or the PRO-seq data themselves. We selected dominant pre-mRNA isoforms using DENR [26] and then stringently masked out possible internal TSSs using dREG [34] and available PRO-cap [33] or GRO-cap [32] data (see Supplementary Fig. S7). We then discarded the first 2250 bp downstream of the TSS—which appeared to include all promoter-proximal pause sites—and the final 250 bp of each TU. Finally, we employed a LOESS-based adjustment to eliminate the “U-shaped” pattern in read depths shared across genes [6], ensuring that they were globally uniform along each gene on average.

We applied our model to the processed data for 6000 robustly expressed protein-coding genes, randomly sampling 2000 genes in each of 10 rounds of analysis and using the variation in these estimates to obtain standard errors for the estimated coefficients κ. We found that the estimates of κ were mostly negative in sign, indicating associations with a reduction in elongation rate (Fig. 3A). The strongest signal, by far, was associated with DNA methylation (κ = −0.20), although its standard error is somewhat large owing to the sparseness of the annotation (at CpG sites only). Moderately strong reductions in elongation rate were also associated with H3K36me3 (κ = −0.095), H3K9me1 (κ = −0.067), and RNA stem-loops (κ = −0.051). Somewhat weaker reductions were associated with both 3′ (κ = −0.033) and 5′ (κ = −0.019) splice sites, CTCF binding sites (κ = −0.022), and other histone modifications (H4K20me1, H3K9me3, H3K4me1; κ ∈ [−0.041, −0.024]). Only two coefficients were positive, indicating associations with increases in elongation rate: those for low-complexity sequence (κ = 0.016) and the H3K79me2 histone mark (κ = 0.030).

These observations are generally consistent with previous observations at the level of entire genes, with negative correlations having been reported for DNA methylation [6, 7], H3K36me3 [6], H4K20me1 [4], splice sites [17, 22, 40], and CTCF binding [17, 19, 21], and positive correlations having been reported for H3K79me2 [6–8] and low-complexity sequences [7]. Our analysis shows, however, that changes in the presence or absence of these features are directly associated with “nearby” changes in elongation rate, further suggesting underlying mechanistic relationships with the movement of Pol II (see “Discussion” section).

To validate our model-based associations, we carried out a simpler, nonmodel-based analysis that compared the average read depths in sites within gene bodies that were annotated (covered) and were not annotated (noncovered) by each feature (Fig. 3B), after a suitable normalization (see “Materials and methods” section). We found that features that had negative coefficients under the model (indicating an association with reduced elongation rate) did indeed show ratios >1 and features that had positive coefficients mostly showed ratios <1 with the exception of H3K79me2, where the effect was weak. The tendency for a local change in read depth could also be observed in “metaplots” of average values across annotated sites (Fig. 3C). Overall, this comparison shows that our model-based analysis does faithfully reflect first-order patterns of relative read depth, but makes some adjustments in rank order—and occasionally in sign—by jointly considering all features together in one unified framework.

We also sought to test the model’s predictions of held-out PRO-seq data, but the predictive power for read counts at individual nucleotide sites is poor, even with simulated data, because the data are so sparse (see Supplementary Fig. S3D and F). We therefore tested the model’s predictions of “pausing locations” within gene bodies, defined as 200-bp intervals having the highest average read counts for each gene (see “Materials and methods” section). We found, even with held-out genes, that model-based predictions of such pausing locations agreed fairly well with the truth (*r*^2^ = 0.68; Fig. 3D). In addition, we tested the model’s predictions of average read counts across larger windows, spanning 1000 bp. At this level of resolution, the predictions for held-out genes were still approximate but much better than for individual nucleotides (*r*^2^ = 0.45; Fig. 3E). These results suggest that the model is effective at getting at underlying elongation rates even if the predictive power for nucleotide-level PRO-seq read counts remains weak.

### An extension to the model accommodates DNA-sequence *k*-mers

In addition to epigenomic factors, it is well known that DNA sequences can also influence local elongation rates, based on studies ranging from bacteria [41] to yeast [42] and mammals [43] (reviewed in [14]). Promoter-proximal pausing in *Drosophila* and mammals is also associated with particular sequence motifs [9, 43, 44]. Recently it was shown using NET-seq (Native Elongating Transcript Sequencing) and PRO-seq data for *Saccharomyces cerevisiae* that nucleotide 5-mers were strongly predictive of local elongation rates, beyond what could be explained by G+C content, DNA folding energy, or sequencing bias [11].

We therefore extended our GLM to consider the *k*-mer content of the local DNA sequence, initially considering 5-mers only (*k* = 5). These 5-mers are accommodated using indicator features in our regression framework (see “Materials and methods” section), which can be used alone or together with epigenomic features. To address the high-dimensionality of the resulting feature vectors, we added a sparsity penalty to our likelihood function, which limits the number of nonzero coefficients and forces the model to choose the 5-mers that are most informative. After experimentation, we settled on an L1 (lasso) penalty and determined the strength of the penalty by cross-validation (see “Materials and methods” section and Supplementary Fig. S8 for details).

We tested this approach with data simulated by SimPol, in which 100 randomly selected 5-mers were assigned negative or positive coefficients and all others were assigned coefficients of zero (see “Materials and methods” section). With the L1 penalty, the model assigned nonzero coefficients also to 100 features, which heavily overlapped the true set (Supplementary Fig. S8). In addition, the predictions of local elongation rate were generally close to the true values (Fig. 4A; *r*^2^ = 0.89). Overall, the method appears to be effective both at recovering 5-mers correlated with elongation rate and at predicting the local elongation rate itself.

### Several *k*-mers are strongly associated with local elongation rates in K562 cells

We re-analyzed the PRO-seq data from K562 cells, this time considering DNA-sequence *k*-mers only. We started with the same 6000 genes as in the epigenomic analysis but this time sampled four batches of 500 genes to limit computational cost. Also, instead of considering 5-mers only, we allowed for *k*-mers of any length up to and including five nucleotides (*k* ∈ {1, 2, 3, 4, 5}). In this version of the model, the *k*-mers of different sizes compete with one another, and the smallest *k*-mer that adequately explains the data will tend to be selected. For example, if the correlations with elongation rate are truly driven by G+C content, the model will tend to choose the G and C 1-mers rather than many different 5-mers containing Gs and Cs. In addition, because the model is additive, larger *k*-mers are assigned coefficients representing their contributions “beyond” those of shorter *k*-mers nested within them. For example, if both G and AG are included in the model, then the coefficient assigned to G will reflect its marginal contribution and the coefficient assigned to AG will reflect only the additional contribution of a preceding A.

When we fitted the model to the K562 PRO-seq data we found that the strongest signals, by far, were for a negative correlation of cytosine (C) nucleotides (κ = −0.24) and a positive correlation of thymine (T) nucleotides (κ = 0.15) with local elongation rate (Fig. 4B). The negative correlation was enhanced when the C was followed by an A (CA) or a T (CT) or when it was preceded by a G (GCA, GCT). The nucleotide trimer AGT was also negatively correlated with elongation rate. The positive correlation of a T was enhanced when it was preceded or followed by additional Ts (TTT, TTG). In some cases, a positive correlation also occurred with As or Gs in place of Ts in the central position (TAA, TAG, AA, TAC, TGG, TGC). The negative correlation with cytosines echoes similar findings in *Escherichia coli* [41] and findings for promoter-proximal pausing in mammals [9, 43], and the positive correlation with A+T-rich sequences is consistent with reports of negative correlations with G+C content [6, 7, 15] with some differences (see “Discussion” section). Interestingly, we found three cases where reverse complementary *k*-mers were associated with fairly strong opposite effects on elongation rate: TGG/CCA, TGC/GCA, and TGT, ACA (positive/negative coefficients in all cases; see “Discussion” section). Altogether, between 159 and 232 *k*-mers were assigned nonzero coefficients across replicates (Supplementary Fig. S9).

As with the epigenomic version of the model, we validated these *k*-mer associations by examining the corresponding relative read depths. We found that the *k*-mers that had negative coefficients did indeed exhibit higher relative read depths, and those with positive coefficients did have lower relative read depths (Fig. 4C). In this analysis, the larger *k*-mers had more divergent relative read depths despite having smaller absolute κ estimates, because, as noted, the κ estimates reflect only the additional contribution associated with the larger *k*-mer context. For example, GCA has higher relative read depth than CA, but the κ estimate for GCA is smaller than for CA. When relative read depths for each significant *k*-mer are plotted along the genome sequence, the local departures from the background levels can be clearly observed (Fig. 4D). By contrast, the relative read depths for insignificant *k*-mers are much less pronounced (Fig. 4E). Interestingly, we found three cases where reverse complementary *k*-mers were associated with fairly strong opposite effects on elongation rate: TGG/CCA, TGC/GCA, and TGT, ACA (positive/negative in all cases). This observation raises the possibility that *k*-mer composition could help promote directionality in Pol II movement.

For comparison with the *k*-mers of various sizes, we also analyzed the data with a version of the model that allowed for 5-mers only. To make sense of the identified *k*-mers, we separately clustered the ones positively or negatively associated with elongation rate and summarized each cluster using a sequence logo (Fig. 4F; see “Materials and methods” section). The clusters negatively associated with elongation rate were clearly dominated by a central C, which tended to be followed by A or T and tended to be preceded by G or A. The positively associated clusters were clearly dominated by Ts with a secondary signal from As.

We also evaluated the performance of the *k*-mer model in predicting pausing locations within gene bodies and held-out read counts in 1-kb intervals (Supplementary Fig. S10A and B). While it performed roughly as well at predicting pausing locations as the epigenomic model (*r*^2^ = 0.64 compared with *r*^2^ = 0.68), its predictions of read counts were substantially better (*r*^2^ = 0.65 compared with *r*^2^ = 0.45).

A possible concern with this analysis is that the *k*-mer associations we find with elongation rate, which reflect nucleotide preferences at the 3′ ends of aligned PRO-seq reads, might be influenced by biases in the PRO-seq protocol. Several lines of evidence, however, suggest that such biases are not driving our results, including comparisons of the bulk distribution of 3′ nucleotides across datasets (Supplementary Fig. S11A and B), an analysis of possible ligation biases (Supplementary Fig. S11C–E), and analyses with a version of the model that explicitly allows for 3′ nucleotide biases (see “Materials and methods” section and Supplementary Fig. S10C). It is also notable that a strong preference for cytosines at sites of promoter-proximal pausing has been noted with NET-seq data as well [9], and the NET-seq protocol does not include the biotin run-on step of PRO-seq (see [31]). Overall, while we cannot rule out some influence from the protocol on our *k*-mer associations, these findings suggest that any such bias should be minimal (see “Discussion” section).

### Most associations with local elongation rate are shared across cell types

We carried out a similar analysis of PRO-seq datasets from three other cell types: CD14+ [26], MCF-7 [28], and HeLa-S3 [27] cells. These datasets were generated by three different laboratories using slightly different methods, making them also informative about the sensitivity of our conclusions to variations on the PRO-seq protocol. For comparison with our results for K562 cells, we analyzed them separately with the epigenomic and *k*-mer versions of our model.

We found that the epigenomic correlates were generally fairly consistent across cell types, with the exception of DNA methylation, which showed a strong negative correlation with local elongation rate in K562 and MCF-7 cells, but a strong positive correlation in CD14+ and HeLa-S3 cells (Fig. 5A). On further inspection, however, we found that this difference traced back to secondary TSSs within TUs, which could not be efficiently identified and removed in CD14+ and HeLa-S3 cells, for which no GRO-cap or PRO-cap data were available. The high degree of sensitivity of the DNA methylation coefficient to internal TSSs was supported by two follow-up analyses: one in K562 cells showing that the sign of the DNA-methylation coefficient changes depending on whether or not the filter for internal TSSs is applied (Supplementary Fig. S12); and one in all four cell types showing a similar effect when H3K4me3 is used as an approximate indicator of internal TSSs in cases where GRO-cap or PRO-cap is unavailable (Supplementary Fig. S13). Both of these analyses suggest a strong dependency of the analysis on DNA methylation signals at internal TSSs.

Aside from this dependency, however, the DNA methylation signal appeared to be a robust predictor of elongation rate and to explain many differences between cell types. For example, we examined ∼16 500 sites that showed high-confidence differences in DNA methylation between K562 and MCF-7 cells, and found that the model did indeed predict highly significant differences in predicted elongation rate at these sites (Supplementary Fig. S14).

The other epigenomic covariates showed much better agreement across cell types. Once DNA methylation was excluded, the κ estimates for K562, CD14+, and HeLa-S3 were all strongly correlated, with pairwise *r* > 0.85 in all cases (Supplementary Fig. S15). The MCF-7 dataset, however, showed somewhat weaker correlation with the others (*r* ≈ 0.7 with K562 and HeLa-S3, *r* = 0.43 with CD14+). We also observed a few other, more minor, differences in epigenomic associations as detailed in the “Materials and methods” section.

We also examined how well a model trained on data for one cell type would predict PRO-seq data from another cell type. In particular, we compared the performance of two models in predicting PRO-seq read depths for K562 cells: one trained on (nonoverlapping) K562 data and one trained on data for MCF-7 cells. We found that these models performed similarly, with only a slight advantage for the model trained on data from the same cell type (Supplementary Fig. S16), indicating that the models are effectively capturing general features of the relationship between epigenomic covariates and elongation rates.

Finally, we fitted a version of the model to NET-seq data for K562 cells from reference [10] and compared it with the model fitted to PRO-seq data for the same cell type. We found close agreement overall in the epigenomic coefficients estimated for the two types of NRS data (Supplementary Fig. S17). The main exception was the coefficient for H3K79me2 marks, which was strongly negative for NET-seq data but weakly positive for PRO-seq data. These two types of NRS data do have somewhat different properties, however, with PRO-seq capturing only transcriptionally active Pol II and NET-seq capturing other Pol II-associated complexes, creating spikes specific to NET-seq data that could explain some differences in these associations [45, 46].

With the *k*-mer version of the model, we also observed general agreement across cell types, with pairwise *r* values of ∼0.8 or greater in all cases (Supplementary Fig. S15B). Among the most prominent *k*-mers (Fig. 5B), the most striking difference was in the κ estimate for cytosines, which in CD14+ and MCF-7 cells was about half that in K562 cells, and in HeLa-S3 cells was nearly cut to zero (possibly for technical reasons; see “Materials and methods” section). Nevertheless, the estimates for cytosines in CD14+ and MCF-7 were still among the largest (in absolute value) κ estimates for those cell types, indicating that cytosines do seem to be associated with a substantial reduction in elongation rate across cell types, even if the strength of association may be sensitive to the details of the PRO-seq protocol (see “Materials and methods” section).

We also wondered whether *k*-mers upstream or downstream of the active site might be correlated with elongation rate, e.g. owing to the energetics of the DNA–RNA hybrid, Pol II–nucleic acid interactions, or structure in the nascent RNA. We therefore applied the *k*-mer model to the datasets for all four cell types, but this time we considered 5-mers that were shifted upstream (in the 5′ direction) or shifted downstream (past the nascent RNA) by 5, 10, 15, 20, or 25 nt. Upstream of the active site, the *k*-mers positively correlated with elongation rate were dominated by A+T-rich sequences, but farther upstream, they included As and Ts in roughly equal proportions, whereas closer to the active site, they were clearly dominated by Ts (Fig. 5C and Supplementary Fig. S18). By contrast, the upstream *k*-mers negatively correlated with elongation rate—particularly at −5 nt—showed a distinctive enrichment for A and G in alternating patterns reminiscent of GAGA-factor binding sites, which are known to be associated with promoter-proximal pausing of Pol II [40, 44, 47]. Downstream of the active site, both the positively and negatively correlated *k*-mers were much less well-defined (Supplementary Fig. S19).

### Predicted local elongation rates are available as a UCSC Genome Browser track

The separate epigenomic and *k*-mer versions of the model both exhibited good predictive performance on held-out PRO-seq data (Fig. 3D and E and Supplementary Fig. S10A and B), but we wondered if performance could be improved by combining all features into one model. We therefore devised a version of the model with all 12 epigenomic features and the *k*-mer features of sizes 1–5, applying the lasso penalty to induce sparsity. We fitted this model to the PRO-seq data for K562 cells and tested it on held-out data, as in the previous experiments. We found that the combined model did perform better than the two separate models, but the improvement relative to the *k*-mer-only model was slight (Supplementary Fig. S20). This result suggests that most of the information in the epigenomic model can be extracted from the *k*-mer composition of the underlying DNA sequences, and overall, the *k*-mers are more predictive than the epigenomic features, perhaps owing to their much denser coverage along the genome.

**Figure 6.**
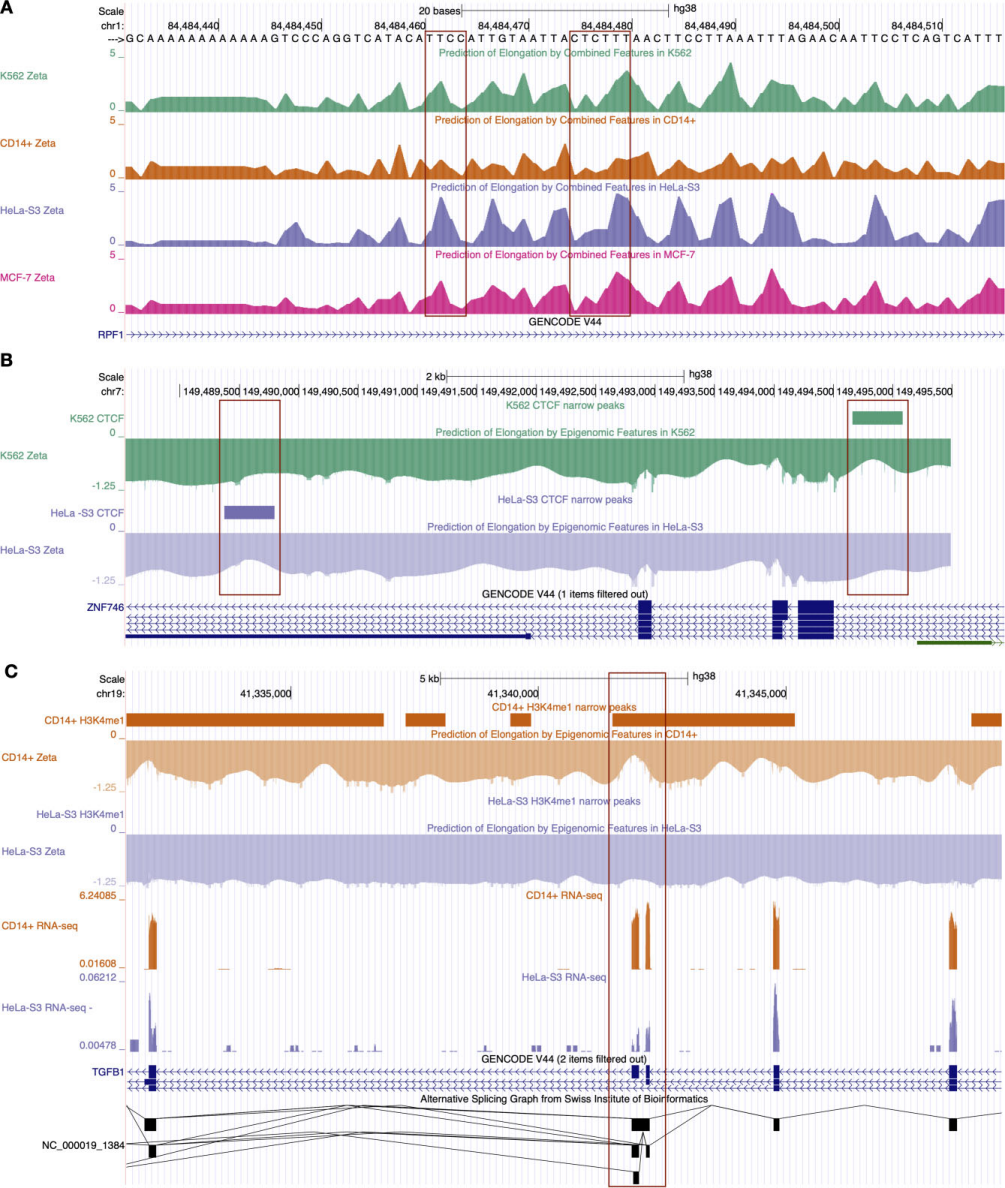
Examples of UCSC Genome Browser tracks showing predicted local elongation rates. (**A**) Predicted local elongation rates based on the combined *k*-mer and epigenomic model for the K562, CD14+, HeLa-S3, and MCF-7 cell types in a region of the *RPF1* gene. Elevated predicted rates at poly-T sequences and reductions at cytosines are consistent across cell lines (boxes). (**B**) Predicted local elongation rates based on the epigenomic model for K562 and HeLa-S3 in a region of the *ZNF746* gene. Cell-type-specific reductions in rates associated with CTCF binding sites are highlighted (boxes). (**C**) Predicted local elongation rates based on the epigenomic model for CD14+ and HeLa-S3 in the alternatively spliced *TGFB1* gene. H3K4me1 presence correlates with rate reduction, accompanying exon inclusion in CD14+ (box). Conversely, its absence correlates with no reduction in rates, accompanying exon skipping in HeLa-S3, as supported by RNA-seq data. In all panels, positive values represent rates on positive strands, while negative values indicate rates on negative strands.

Based on this combined model, we created a UCSC Genome Browser track (available at http://compgen.cshl.edu/elongation-rate-tracks.php) showing the predicted nucleotide-specific local elongation rates genome wide (Fig. 6). This track allows our model-based predictions to be viewed alongside gene annotations, epigenomic data, and many other types of genomic data. In this browser track, each of the four cell types (K562, CD14+, MCF-7, and HeLa-S3) is configured as a complete composite track, enabling users to easily select cell types and models as needed. The track reveals that elongation rate patterns across cell types are in general similar, reflecting both similar sequence-related effects and dependencies on shared epigenomic marks (Fig. 6A and Supplementary Fig. S21A). In some cases, however, interesting differences between cell types can be observed, such as predicted decreases in elongation rate owing to cell-type-specific CTCF binding sites (Fig. 6B) or H3K4me1 histone marks (Supplementary Fig. S21B). In other cases, cell-type-specific epigenomic marks and associated decreases in local elongation rate appear to be linked to cell-type-specific exon-inclusion events (Fig. 6C and Supplementary Fig. S21C). This browser track is publicly available either for browsing or for download of raw data.

### A CNN delivers improved predictive performance

We wondered if predictive performance could be improved further by using a neural network in place of our GLM, allowing nonlinear and feature-interaction relationships with local elongation rate to be captured. We also reasoned that the use of a CNN might allow larger DNA-sequence contexts to be considered, as has been shown in regulatory genomics [48–50]. To test this idea, we used PyTorch to implement a relatively simple CNN model that considers 100 bp of both DNA-sequence and epigenomic context, with three CNN layers for the sequences and one for the epigenomic data (see “Materials and methods” section and Supplementary Fig. S22). To allow this model to be easily compared with the GLM, and to ensure that similar predictions of the nucleotide-specific relative elongation rate ζ_*i*, *j*_ could be extracted from it, we used an output layer and loss function based on the GLM (i.e. with output representing the local elongation rate and loss equal to the negative log likelihood of the read-count data under the Poisson model; see “Materials and methods” section). We trained the model to minimize this loss on real PRO-seq data, similar to the procedure with the GLM.

**Figure 7.**
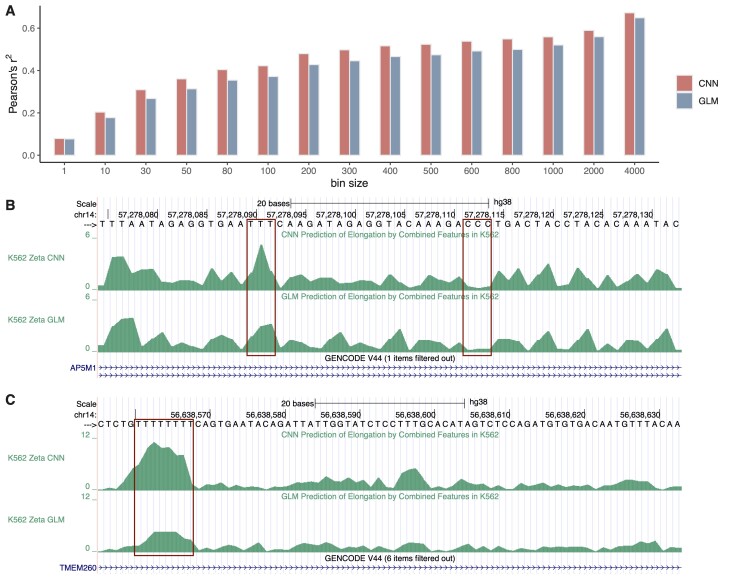
Prediction of elongation rates using a CNN based on combined DNA-sequence and epigenomic data. (**A**) Comparison of predictive performance of the CNN with the combined epigenomic and *k*-mer GLM. Performance is measured by Pearson’s *r*^2^ between the predicted and true PRO-seq read depths (*X*_*i*_) at various window (bin) sizes, using held-out data for K562 cells. (**B**) UCSC Genome Browser tracks showing predicted local elongation rates for the CNN and GLM for K562 cells in a region of the *AP5M1* gene. Under both models, rate increases are observed at poly-T sequences and reductions are observed at cytosines (boxes). (**C**) Similar comparison of tracks in a region of the *TMEM260* gene showing somewhat exaggerated variability in the CNN, particularly in poly-T sequences (box).

We found that the CNN did achieve somewhat lower loss in training than the GLM and did perform significantly better in predicting held-out PRO-seq data. In particular, when we used the CNN to predict held-out PRO-seq read counts in windows of various sizes, we observe improvements in Pearson’s *r*^2^ of 5%–15%, with the largest improvements in windows of 10–50 bp and a top performance of *r*^2^ = 0.67 in 4-kb windows (Fig. 7A). To see if the CNN was making use of the same sequence patterns as the GLM, we compared the predicted local elongation rates ζ_*i*, *j*_ under the two models at sites having *k*-mers assigned nonzero coefficients by the GLM. We found, indeed, that the predictions at these sites were strongly correlated, particularly for 3-mers (*r*^2^ = 0.83) but also for 4-mers (*r*^2^ = 0.77) and 5-mers (*r*^2^ = 0.70), indicating that the two models are driven by similar sequences (Supplementary Fig. S23). An attribution analysis based on Captum’s GradientShap implementation (see arXiv: https://doi.org/10.48550/arXiv.1705.07874), however, showed that the CNN draws information not only from the 3–5 nt centered on the active site, as does the GLM, but from as many as seven flanking bases on each side (Supplementary Fig. S24). Altogether, we find, in comparison to the GLM, that the CNN discovers similar sequence associations but is able to improve performance by taking advantage of weaker signals in flanking sites.

We added the CNN-based predictions as subtracks in our UCSC Genome Browser track, where they can easily be compared with the GLM-based predictions. It is evident from these tracks that the two sets of predictions are generally similar (Fig. 7B), but the CNN-based predictions display a somewhat larger dynamic range (Fig. 7C). We recommend the use of the CNN-based tracks when maximum predictive performance is needed. Because the improvement over the GLM is modest, however, and the CNN-based predictions are more difficult to interpret, we have based our main analysis on the GLM. In future work, we plan to explore other neural-network architectures and improve these predictions further.

## Discussion

In this article, we introduce a new probabilistic model for evaluating correlations between local elongation rate and a wide variety of genomic and epigenomic features. Our model explains nucleotide-specific NRS read counts by assuming they are Poisson-distributed with mean inversely proportional to the local elongation rate, which in turn is determined by an exponentiated linear function of nearby genomic and epigenomic features. We separately applied epigenomic and DNA *k*-mer versions of the model to data for four mammalian cell types. DNA methylation emerged as the strongest epigenomic correlate of local elongation rate (although one that is sensitive to filters for internal TSSs), followed by H3K36me3 and H3K9me1 histone marks and RNA stem-loops, all of which were associated with slow-downs of Pol II. Other significant negative correlates of rate included splice sites, CTCF binding sites, and several other histone marks. Low-complexity sequences and H3K79me2 marks were positively associated with elongation rate. In our DNA *k*-mer analysis, the strongest signals came from cytosines, which were associated with reductions in elongation rate, and thymines, which were associated with increases in elongation rate. Notably, the effects of cytosines and DNA methylation are not confounded in our modeling framework but can be distinguished from one another, owing to our strategy for encoding methylation (see “Materials and methods” section). We also showed that it is possible to improve the predictive performance of the model somewhat by replacing the GLM with a CNN, which allows for nonlinearity, interactions between features, and larger sequence contexts, at some cost in interpretability. We have used our GLM and CNN models to generate a publicly available UCSC Genome Browser track for the K562, CD14+, MCF-7, and HeLa-S3 cell types. Work is underway to further improve our CNN models.

A key feature of our model is that it directly predicts a “local”, nucleotide-specific elongation rate from features at, or within a few bases of, each nucleotide site. By contrast, most previous studies have focused on correlations at the level of entire genes. A disadvantage of our approach is that, because it is based on a single timepoint and assumes Pol II occupancy is at steady state, it cannot estimate absolute elongation rates. On the other hand, it provides high-resolution predictions of the relative local rate, and it identifies covariates of rate whose presence or absence is physically proximal to changes in NRS read depth. This physical proximity increases the likelihood that these correlations reflect mechanistic, causal relationships—although we still cannot prove causality in this setting.

The correlations our model revealed were generally consistent with previous reports. For example, several studies have identified negative correlations of elongation rate with DNA methylation in mammalian cells [6, 7] (see also [12]). In addition, there have been reports of positive correlations with H3K79me2 [6–8] as well as negative correlations with H3K36me3 [6] and H4K20me1 [4]. Notably, some of the negative signals associated with histone marks (with the exception of H3K79me2) may simply reflect the presence of nucleosomes, which are not separately accounted for by our model or other reported results. Elongation rates are now well known to be negatively correlated with exon density [6, 7, 17], and this relationship appears to be driven, at least in part, by a local reduction in rate at splice sites [17, 22, 40], likely from co-transcriptional splicing. A negative correlation of elongation rate with CTCF binding has also been noted, which in some cases influences splicing [17, 19, 21]. The relationship between RNA secondary structure and elongation rates does not appear to have been examined genome-wide in mammals, but associations of such structure with reduced elongation rates have been observed in bacteria, yeast, and *in vitro* systems [17, 51–53]. A positive correlation of elongation rate with low-complexity sequences has also been reported [7]. Our analysis shows that these correlations hold at the local level as well as at the level of averages across genes.

In addition, previous studies have generally considered each potential covariate separately, whereas our model combines them in a unified framework, in such a way that their relative contributions to elongation rate can be compared. As a result, we can say, e.g., that the impact of DNA methylation on elongation rate is about twice that of H3K36me3 marks in K562 cells, accounting for all other covariates (Fig. 3A). Perhaps owing to our joint model, we do see some minor differences from previous results; e.g. H4K20me1 [7] and H3K4me1 [6] have been reported to be positively correlated with elongation rate, whereas we find negative correlations. In an analysis of this kind, the directionality of a relationship can change depending on whether or not other correlated covariates are considered, as was observed with H3K79me2 in Fig. 3B.

The negative correlation we observe with a cytosine at the 3′ end of a nascent transcript echoes a similar finding for promoter-proximal pausing, where paused Pol II shows a strong preference for cytosines at the active site [9, 43]. These studies have also shown some enrichment for G at the preceding position, as in our findings, although the motifs they identified were generally more dominated by Cs and Gs than ours. A similar, but weaker, preference for cytosines was also previously observed outside of the promoter-proximal region based on CoPRO-cap and PRO-seq data [31]. This preference for cytosines has been conjectured to be a consequence of cytosine being the least abundant nucleotide and therefore the slowest to incorporate into nascent transcripts [31]. A similar association between cytosines and pausing of RNAP has been observed in *E. coli*, where it appears to result from RNAP–nucleic acid interactions that inhibit next-nucleotide addition [41] (see also [54]). In this case, however, the preference is for either C or T, both of which tend to be followed by G.

The positive correlation we find between A+T-rich sequences and elongation rate is consistent with many reports indicating a general correlation with G+C content, with slower elongation rates in G+C-rich sequences—and, accordingly, faster rates in A+T-rich sequences—likely resulting from stronger RNA–DNA hybrids and a tendency to form stable RNA secondary structures [6, 7, 15]. It has also been reported that increased elongation rates in A+T-rich introns are stimulated by the U1 small nuclear RNP at 5′ splice sites [55]. Our findings differ from these previous reports, however, in indicating that the effect seems to be driven by thymine somewhat more than adenine bases. Another finding from our study, which appears to be new, is that certain *k*-mers and their reverse complements have opposite associations with elongation rate. As pointed out by an anonymous reviewer of this work, this observation raises the intriguing possibility that *k*-mer composition could help promote directionality in Pol II movement, which may be worth exploring in future work.

Our analysis assumes that Pol II occupancy along the genome is at a steady-state equilibrium among the cells in the sample, a situation that seems likely to be reasonably approximated in cell lines that have not been subjected to a treatment or stimulus (control samples). The averaging effect of sequencing a pool of cells should help further in establishing a reasonable proxy for such an equilibrium. Importantly, by operating under this steady-state paradigm, we are able to analyze all expressed genes, not just a subset at which expression can be induced, as in strategies that measure elongation rates from time-course data (e.g. [5–7]). We also avoid the off-target effects of chemical treatments that block initiation or pause escape. Nevertheless, it may be worthwhile, in future work, to extend our GLM for transcription elongation to the nonequilibrium setting and see whether new correlates of elongation rates can be detected from time-course data.

A second, perhaps more delicate, assumption is that NRS read counts faithfully reflect the density of transcriptionally engaged polymerases across the genome. The main concern here is that read counts could be influenced by biases in library preparation, sequencing, read mapping, or other processes. In principle, any genomic or epigenomic feature favored or disfavored in these processes could spuriously appear to be associated with elongation rate in our analysis. Most of our epigenomic associations should be fairly robust to such biases, since they reflect modifications to the DNA template rather than the nascent RNA. RNA stem-loops are a possible exception, but our findings suggest that they are overrepresented in PRO-seq read counts, rather than underrepresented, as might be expected from interference in capturing structured RNAs.

On the other hand, it is easy to imagine biases that would affect the *k*-mer associations that we detect, particularly the single nucleotides that we find to be overrepresented (C) or underrepresented (T) at the 3′ ends of aligned reads. In several follow-up analyses, however, we could find no evidence that biases in the protocol were responsible for the cytosine and thymine associations with elongation rate. First, we found that the bulk distribution of 3′ nucleotides for PRO-seq reads was fairly consistent across datasets, even when generated by different research groups (Supplementary Fig. S11A and B), suggesting that it is not highly sensitive to variations of the protocol. Second, we found that the cytosine enrichment and thymine depletion were present with or without removal of polymerase chain reaction duplicates. Third, we tested directly for a ligation bias, despite that T4 RNA ligase has been reported to have little sequence bias [56]. Specifically, we made use of adapter sets in which the 5′ adapter incorporates a UMI with randomly occurring nucleotides at its 3′ end, and compared adapter dimers that contained inserts with ones that did not, finding no difference in their cytosine frequencies and little difference overall (Supplementary Fig. S11C–E). Finally, we devised a “sequence-biased” version of the model that expects the 3′-most base to appear in proportion to its bulk distribution, under the assumption that an unknown bias drives its frequency, and fitted it to the data, finding that, while the coefficients for C and T were greatly reduced (by design), the coefficients for most larger *k*-mers were relatively unaffected (see “Materials and methods” section and Supplementary Fig. S10C). Altogether, we could find no aspect of the protocol that could explain our associations with C, T, or other *k*-mers. It is also worth noting that NET-seq data—which is produced using a quite different protocol from PRO-seq, without run-on—also shows a strong preference for cytosines at sites of promoter-proximal pausing [9].

To our knowledge, this study represents the first attempt to model local rates of transcription elongation across mammalian genomes. Overall, we find that many features that correlate with elongation rates at the level of entire genes do appear to result in local changes to the elongation rate along gene bodies. By considering these features together in either a unified probabilistic model or a CNN, we can obtain fairly accurate predictions of the local rate, as indicated by our simulations and tests with held-out data. We have made predictions for four cell types publicly available in a UCSC Genome Browser track. We anticipate that they will be useful in a wide variety of downstream analyses, e.g., by helping to identify potential cases where transcriptional output is regulated through changes in elongation rate or providing hypotheses about mechanistic influences on elongation rate.

## Supplementary Material

gkaf092_Supplemental_File

## Data Availability

Open-source code to estimate model parameters and predict nucleotide-specific relative elongation rates is available on Zenodo with the following DOIs 10.5281/zenodo.14757127 and 10.5281/zenodo.14757102. The predictions for elongation rate are available in a UCSC Genome Browser track at http://compgen.cshl.edu/elongation-rate-tracks.php.
