# Supplementary material for: Probabilistic and machine-learning methods for predicting local rates of transcription elongation from nascent RNA sequencing data
Source: Nucleic Acids Res. 2025 Feb 18;53(4):gkaf092. doi: 10.1093/nar/gkaf092 (PMC11833694; doi:10.1093/nar/gkaf092)
Supplement: gkaf092_Supplemental_File [file gkaf092_supplemental_file.pdf]

Supplementary Information for: Probabilistic and machine-learning methods for predicting local rates of transcription elongation from nascent RNA sequencing data

Lingjie Liu[1,2], Yixin Zhao[1], Rebecca Hassett[1], Shushan Toneyan[1], Peter K. Koo[1], and Adam Siepel[1,2,*]

[1]Simons Center for Quantitative Biology, Cold Spring Harbor Laboratory, Cold Spring Harbor, NY
[2]Graduate Program in Genetics, Stony Brook University, Stony Brook, NY
[*]Corresponding author: asiepel@cshl.edu

Supplementary Figures

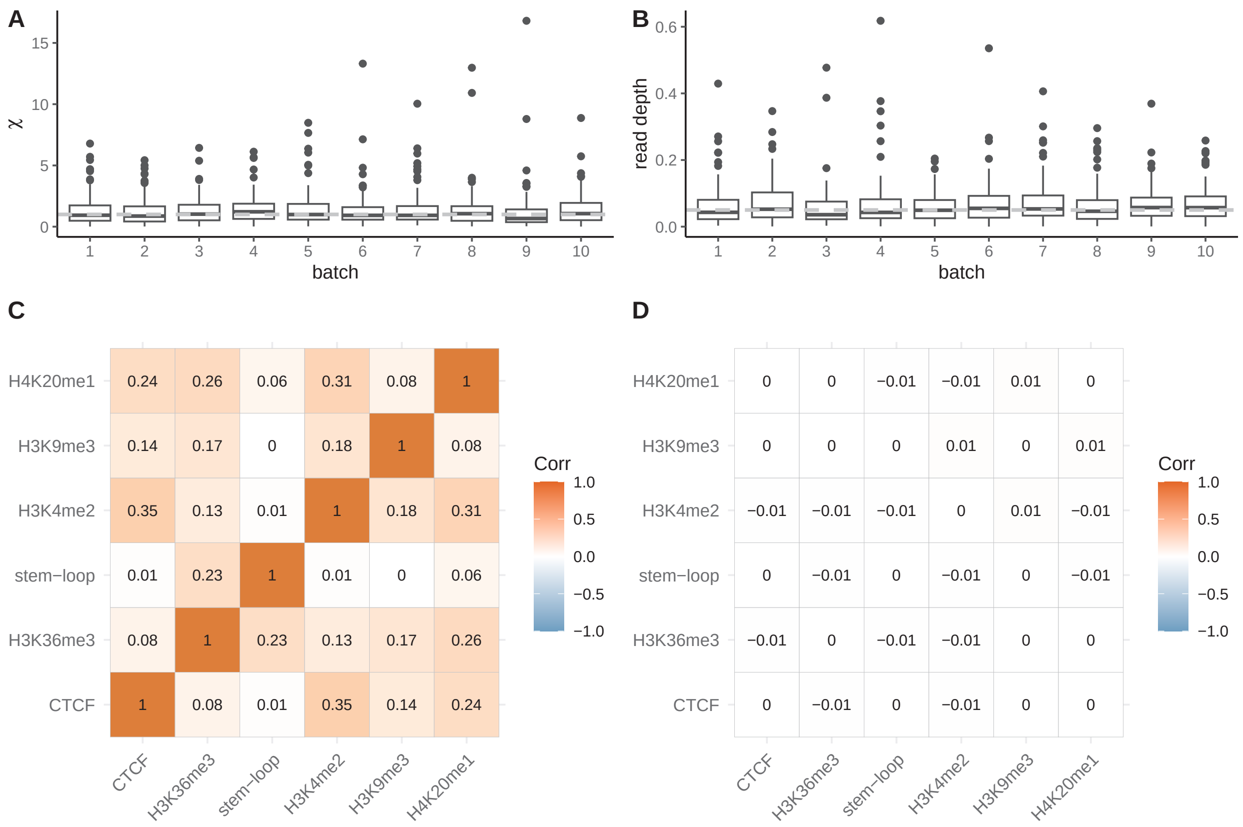

Supplementary Figure S1: **A**. The distribution of the scaled initiation rate χ in ten rounds of simulation, sampled from the estimated χ of real K562 PRO-seq data [1]. The dashed line represents the median of 1. **B**. The distribution of read depth in ten rounds of simulation of synthetic data. The read depth has been adjusted to match that of real K562 PRO-seq data [1], with a median value of around 0.05 [2]. **C**. Correlation map of selected features from real K562 data. **D**. The difference in correlation map of selected features between the sampled covariates for simulations and the real K562 covariates.

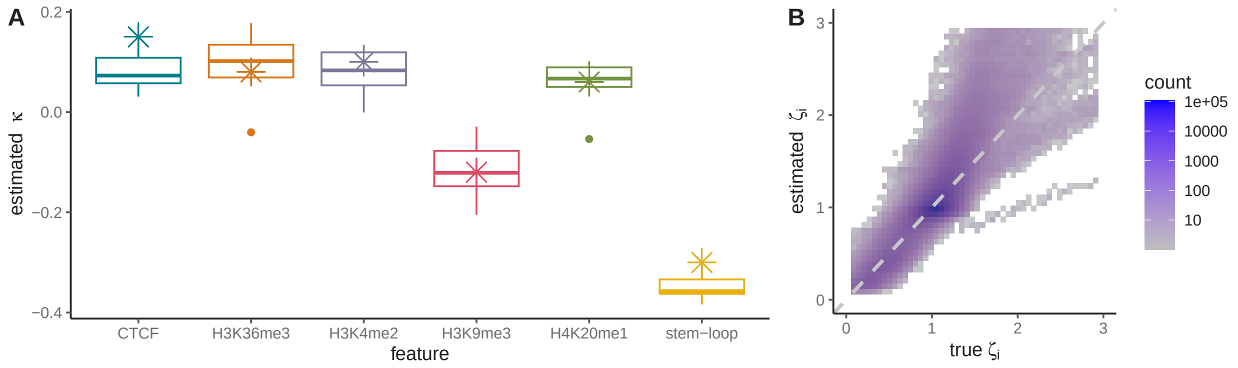

Supplementary Figure S2: **A & B.** The simulation incorporating Gaussian noise with a random assignment of the ground truth κ. To emphasize the robustness of the inference to departures from our initial experiment, we selected randomly sampled values that mostly had opposite signs of the κ values estimated from real data. **A**. Box plots for estimated coefficients κ in ten replicates compared with ground truth in simulations (crosses). **B**. Estimated vs. true nucleotide-specific elongation rates ζ_i across all simulated TUs ($r^2 = 0.85$).

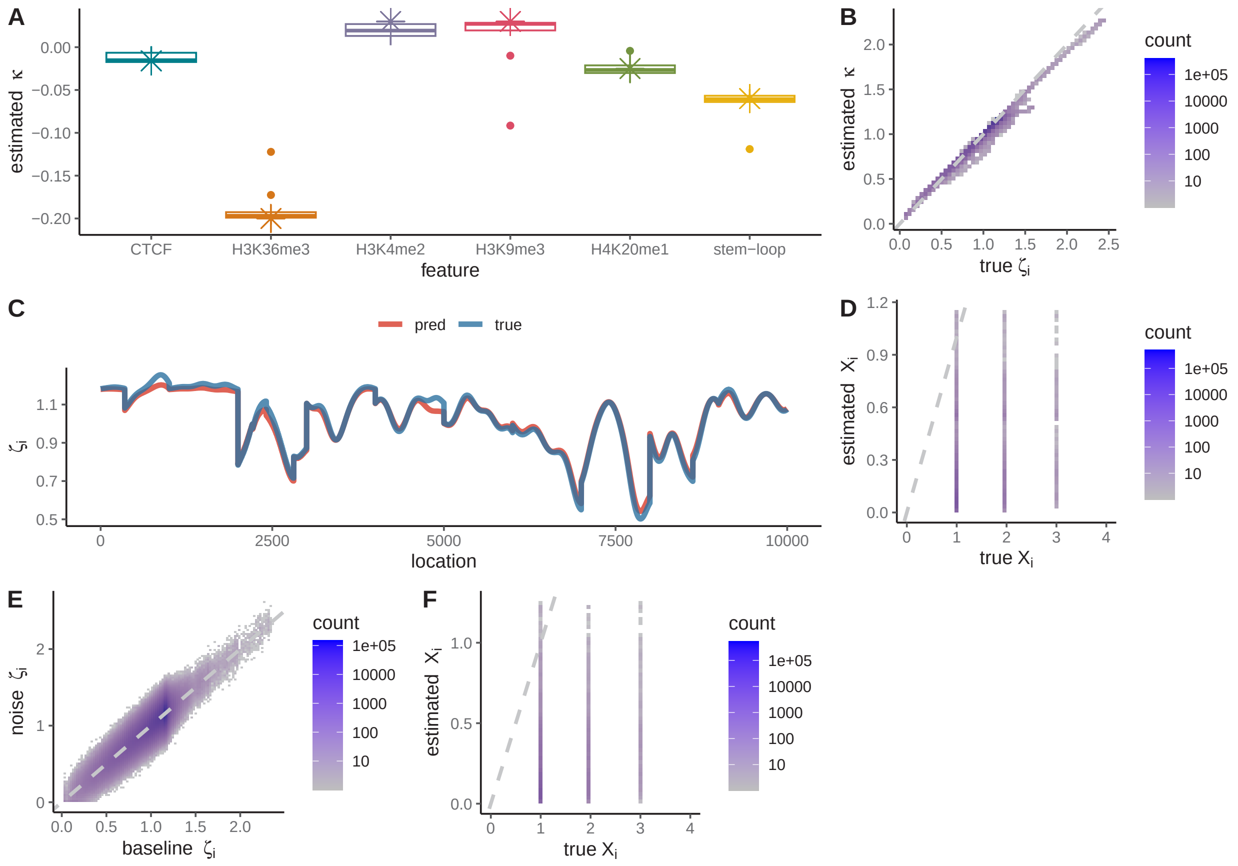

Supplementary Figure S3: **A & B & C & D.** The simulation with an input of baseline ζ_i that is exactly generated by the same generalized linear model used for inference, with no other unmodeled source of variation. **A**. Box plots for estimated coefficients κ in ten replicates compared with ground truth in simulations (crosses). **B**. Estimated vs. true nucleotide-specific elongation rates ζ_i across all simulated TUs ($r^2 = 0.99$). **C**. Estimated vs. true nucleotide-specific elongation rates ζ_i along an individual TU in ten replicates ($r^2 = 0.98$). **D**. Estimated vs. true nucleotide-specific PRO-seq read count X_i across all simulated TUs ($r^2 = 0.067$). **E**. The comparison of baseline ζ_i that is exactly determined by the GLM and the noise ζ_i after introducing Gaussian noise ($r^2 = 0.75$). **F**. Estimated vs. true nucleotide-specific PRO-seq read count X_i across all simulated TUs with an input of noise ζ_i ($r^2 = 0.07$).

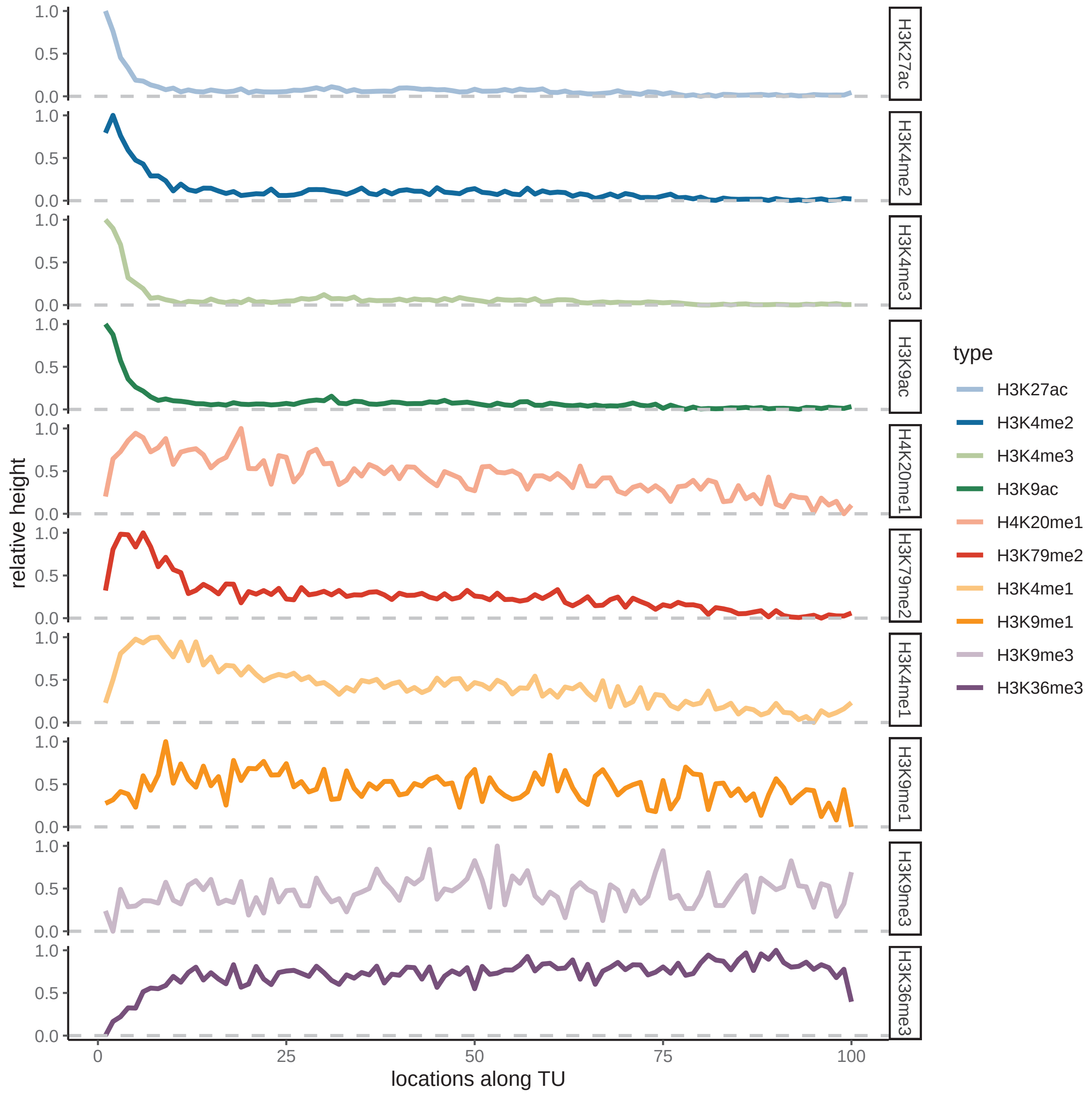

Supplementary Figure S4: Distribution of multiple histone marks from TSSs to the end of gene bodies across genes. To visualize this distribution, the ChIP-seq signals for each histone mark have been scaled from 0 to 1 as relative heights shown on the y-axis.

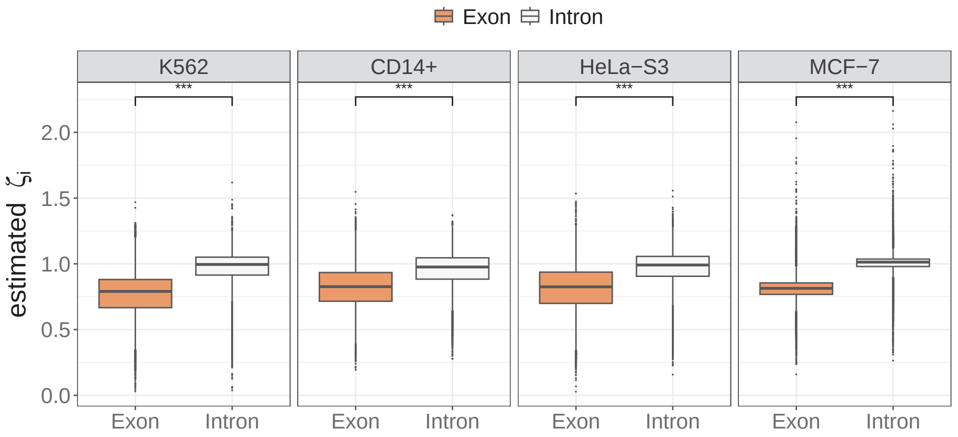

Supplementary Figure S5: Analysis of estimated local elongation rates in exons and introns across approximately 3,000 gene bodies in each of the four cell lines. Asterisks (***) denote t-test p-values < 0.001.

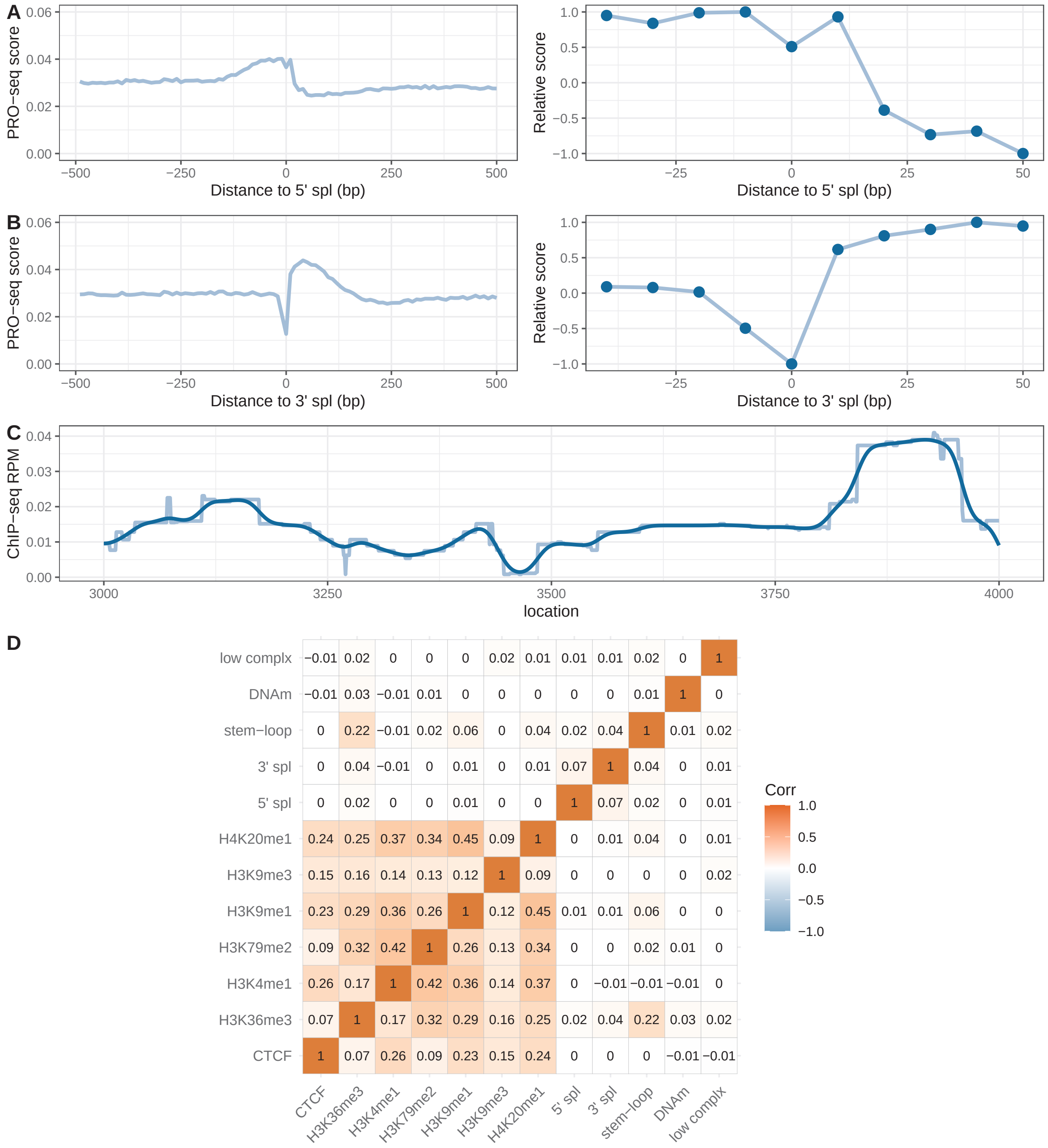

Supplementary Figure S6: **A & B.** Generalized filters were applied to the $5'$ and $3'$ splicing sites. A vector of nonnegative scale factors (*right*) was estimated from metaplots (*left*) of PRO-seq data centered on the feature of either $5'$ or $3'$ splicing sites. **C**. A Gaussian filter was applied to smooth the ChIP-seq-based features. An example of CTCF ChIP-seq signal smoothing is shown. **D**. The correlation map of all epigenomic and annotated features in the actual analysis of K562 cells.

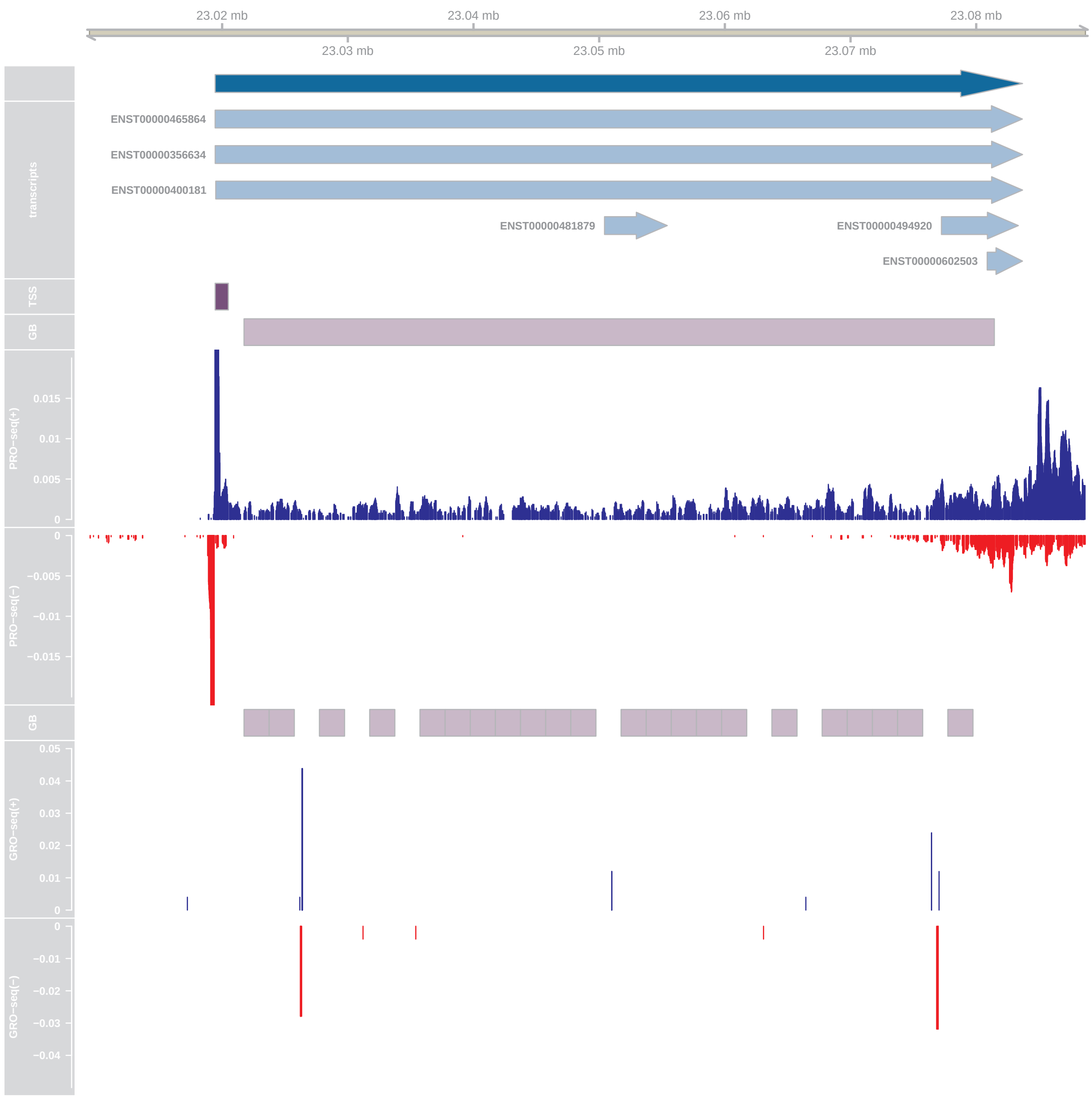

Supplementary Figure S7: The selection of the gene body of the *MFSD4B* gene, refined by several measures. The annotations of the whole gene are colored in dark blue, while the transcript isoforms are colored in light blue. The TSS and the total gene body were identified by DENR with K562 PRO-seq data. Specifically, internal TSSs within gene bodies were masked based on GRO-cap peaks (see **Methods**).

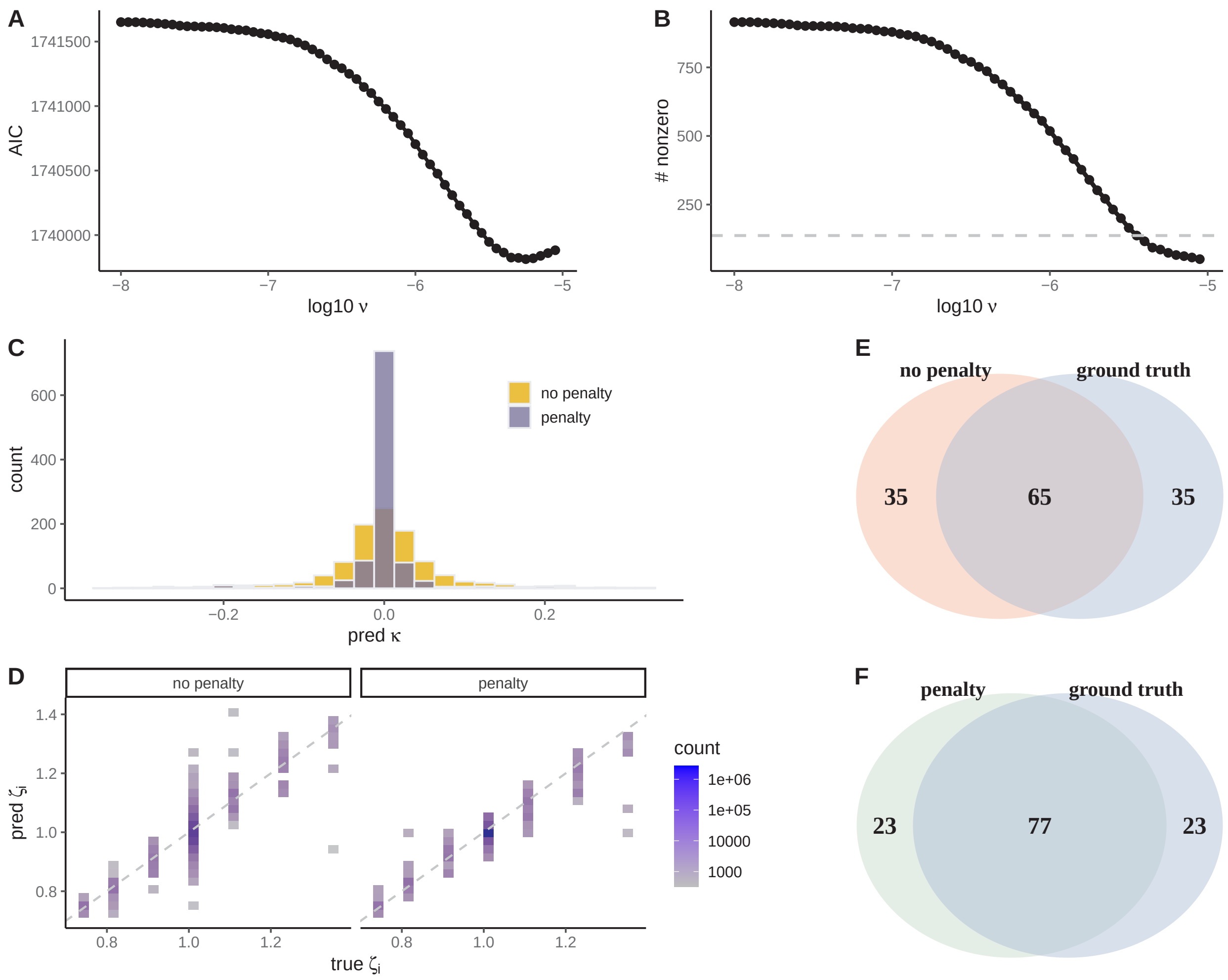

Supplementary Figure S8: Determination of the hyperparameter (ν) of L1 regularization in the simulations for the 5-mer model. **A**. Akaike Information Criterion (AIC) values for held-out testing data with a grid of possible ν values. **B**. The number of non-zero 5-mers by the framework applying L1 regularization. The dashed line represents the number of non-zero 5-mers based on the optimal ν determined by the lowest AIC value. **C**. Successful shrinkage of parameters after the application of penalty, contrasted with settings without penalty. **D**. Predicted vs. true nucleotide-specific elongation rates ζ_i, in settings without penalty (*left*) and with penalty (*right*), with Pearson's r^2 of 0.76 and 0.89, respectively. **E & F**. Comparison of the top 100 5-mers with the most significant coefficients selected by the model and the ground truth in settings without and with L1 penalty, respectively.

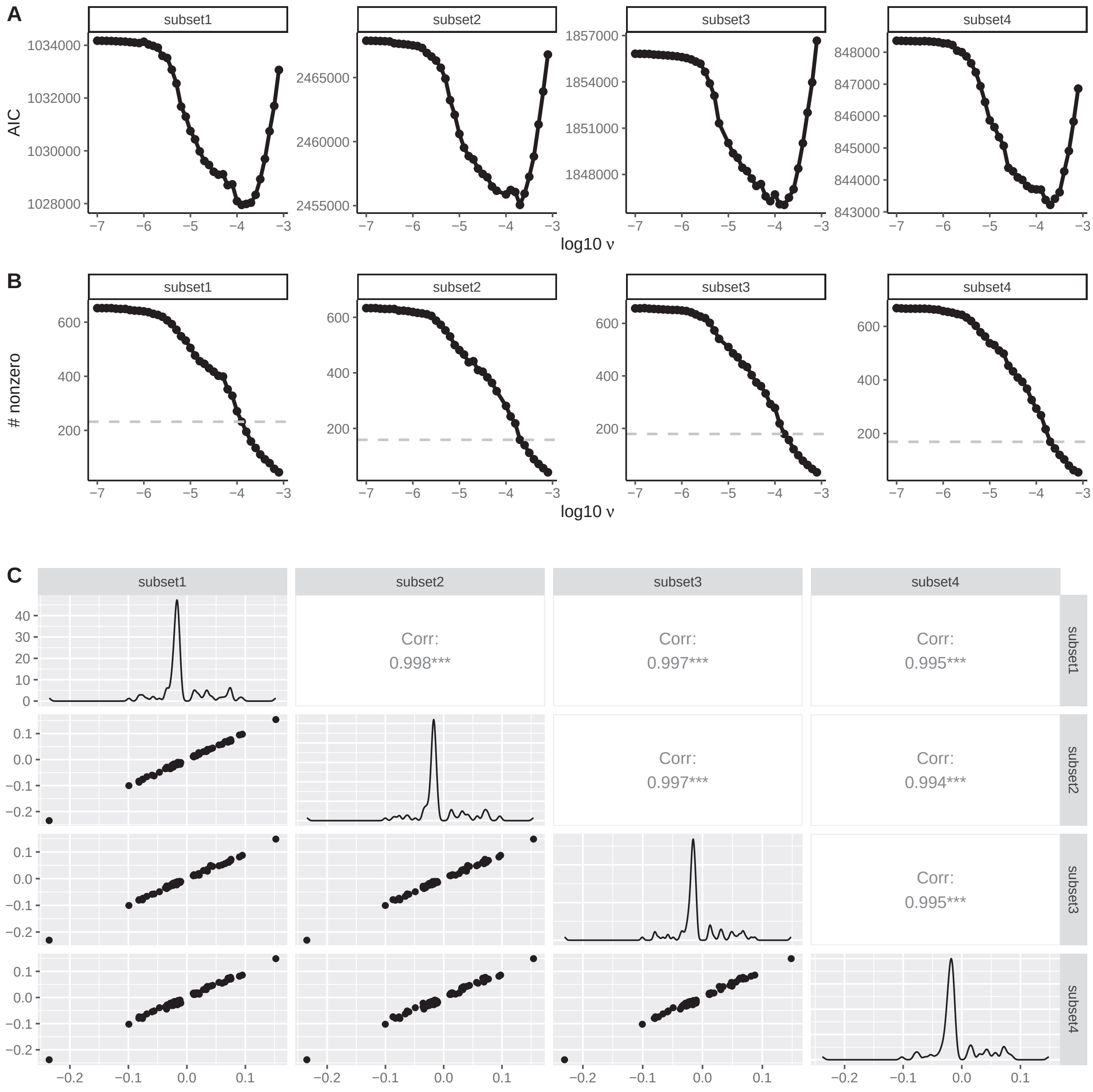

Supplementary Figure S9: The k-mer model in K562 cells. **A**. AIC values for each L1 hyperparameter ν across four sampled batches. **B**. The number of non-zero k-mers by the framework applying L1 regularization across four sampled batches. The dashed line represents the number of non-zero k-mers based on the optimal ν determined by the lowest AIC value. **C**. The consistency of the estimated κ of significant k-mers (N = 105) across four sampled batches.

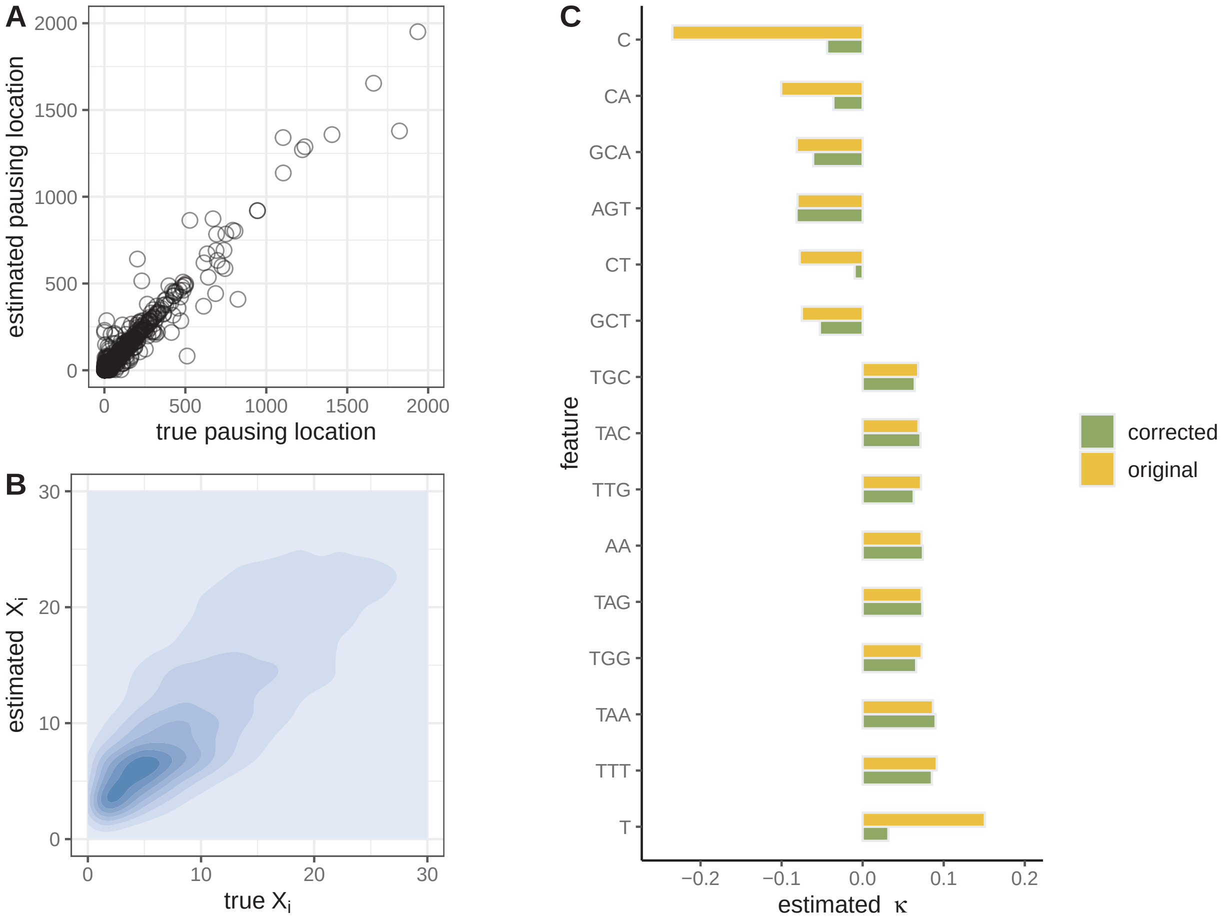

Supplementary Figure S10: **A & B.** The performance of the k-mer model in K562 cells. **A**. Estimated vs. true pausing locations within gene bodies ($r^2 = 0.64$). **B**. Estimated vs. true PRO-seq read depths (X_i) for held-out data averaged over 1kb intervals for all TUs ($r^2 = 0.65$). **C**. Comparison of estimated coefficients κ between two k-mer models: the original model and the extension model allowing for sequence bias (see **Methods**).

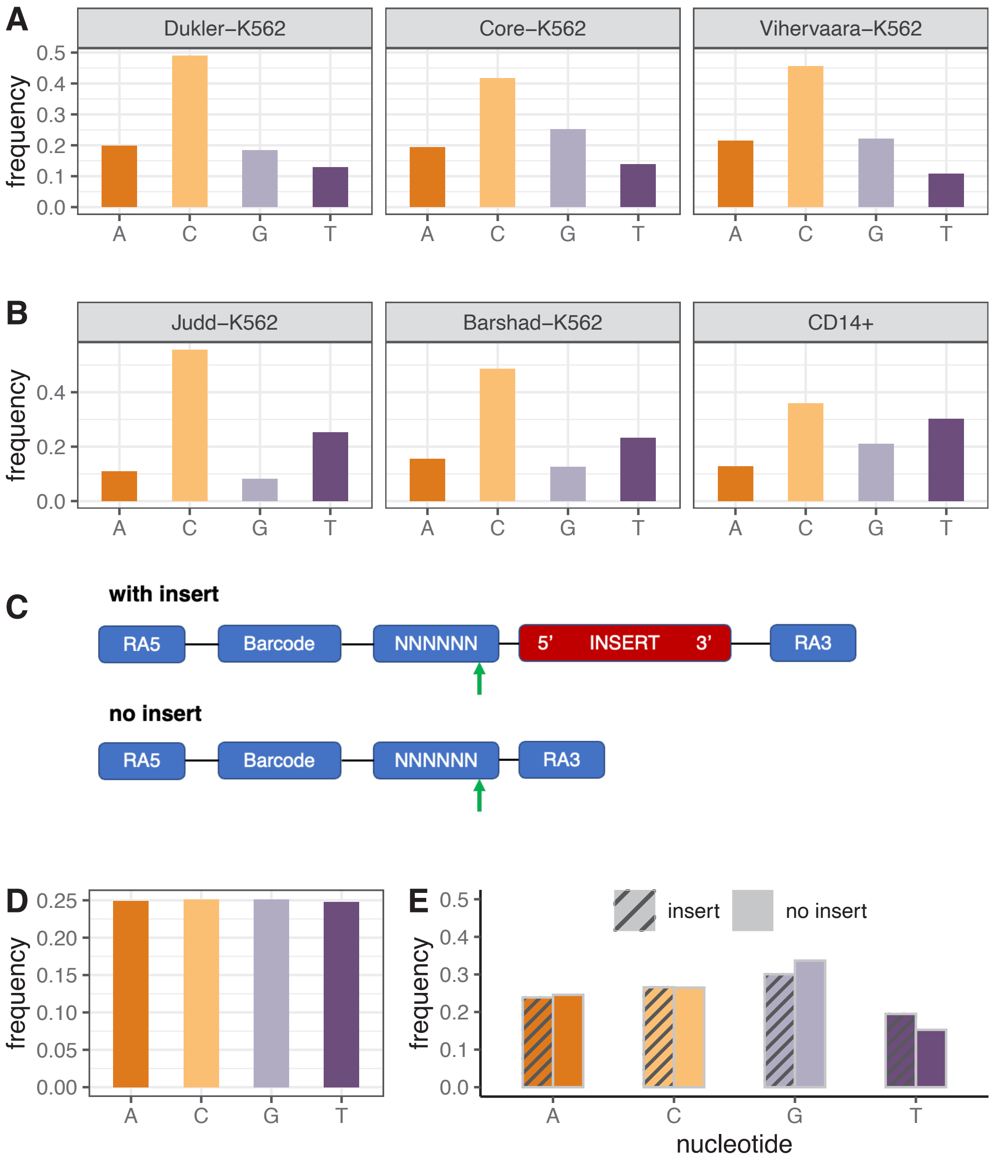

Supplementary Figure S11: **A**. The distribution of the 3′ end base in PRO-seq data from the K562 cell line, acquired through a run-on experiment involving 4 dNTPs with equal concentrations from different research groups. **B**. The distribution of the 3′ end base in PRO-seq data from the K562 and CD14+ cell lines, acquired through a run-on experiment involving 2 dNTPs. **C**. Examination utilizing CD14+ PRO-seq data with a UMI design ligated to an insert. An illustration depicting the structure of reads, either with or without inserts. The green arrow indicates the 3′ end of UMIs. **D**. The distribution of the 3′ end constitution of UMIs in the CD14+ library, with a design to facilitate the examination of potential ligation bias. **E**.The distribution of the 3′ end base of UMIs in sets with or without successful inserts, indicating an absence of ligation bias towards cytosine.

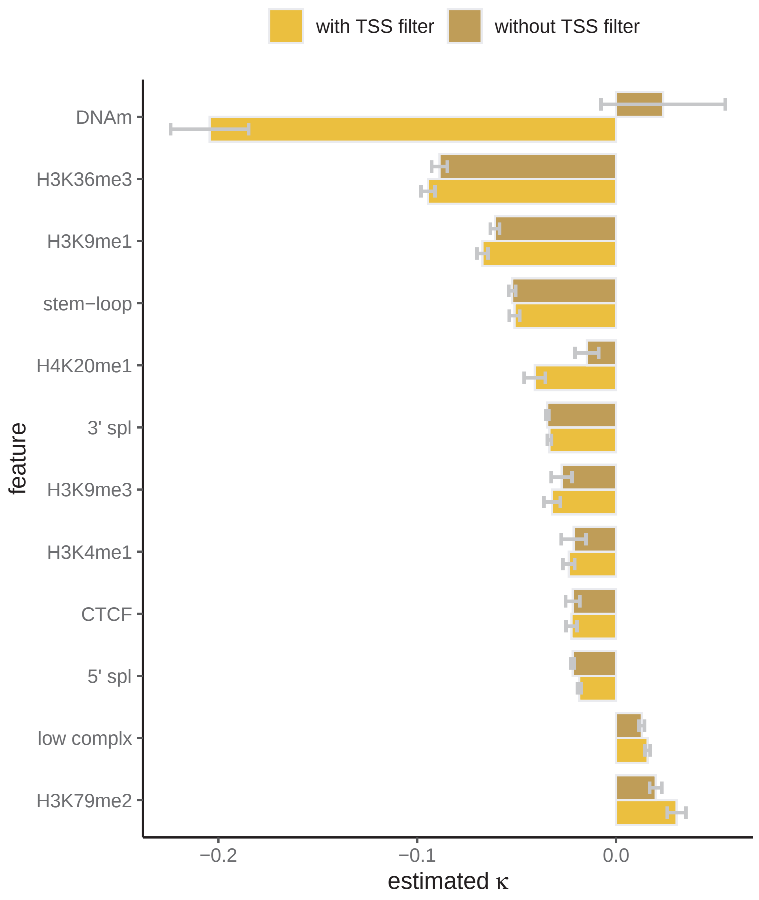

Supplementary Figure S12: Analysis of K562 cells illustrating the impact of internal TSSs on the estimation of DNAm coefficients. In the "without TSS filter" scenario, the entire gene body is included, and in the "with TSS filter" scenario, internal TSSs based on GRO-seq signals are excluded within the same gene body. Error bars represent the standard deviation of rounds of sampled genes. The most notable difference lies in the DNAm coefficient: "without TSS filter" shows a positive correlation between DNAm and elongation rate, while "with TSS filter" shows a negative correlation.

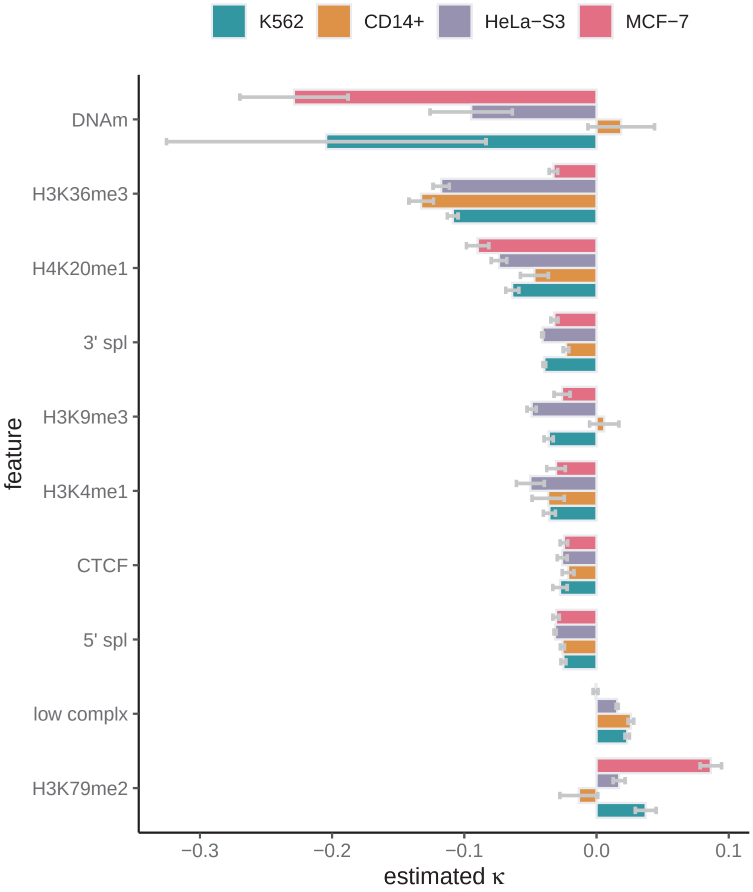

Supplementary Figure S13: Estimated coefficients κ for ten epigenomic features based on PRO-seq data for four cell lines with internal TSS filters applied. H3K4me3 ChIP-seq signals were used as filters for HeLa-S3 and CD14+ cells, where PRO-cap or GRO-cap data was unavailable, while GRO-cap and PRO-cap signals were used for K562 and MCF-7 cells, respectively. As shown, the H3K4me3 ChIP-seq filter produces a similar effect to filters based on PRO-cap and GRO-cap data, causing the coefficient κ for DNAm to change from positive to negative in HeLa-S3 cells. In CD14+ cells, this filter does not change the sign of the DNAm coefficient κ but it does drive it nearly to zero.

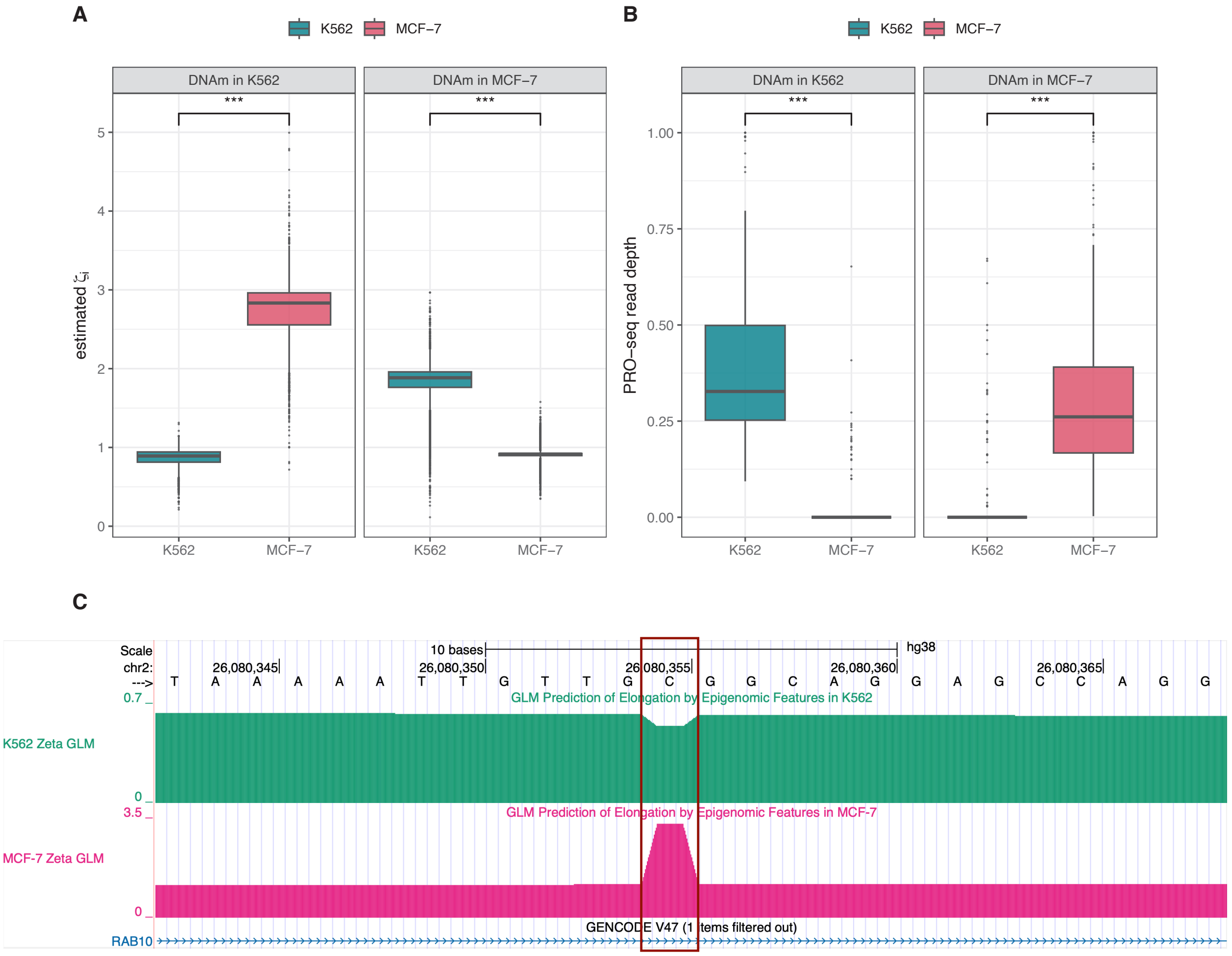

Supplementary Figure S14: Validation of cell-type-specific elongation rate predictions using differential DNA methylation sites in K562 and MCF-7 cells. Cell-type-specific WGBS data was used to identify CpG sites with differential methylation. CpG sites were required to have WGBS coverage in both cell types, with a score of 100 in one cell type and 0 in the other. This stringent approach identified approximately 16,500 high-confidence CpG sites within gene bodies that differed in methylation status between K562 and MCF-7 cells. **A.** Comparison of predicted nucleotide-specific relative elongation rates ζ_i at differential DNA methylation sites between two cell types. As expected, the predicted local relative elongation rate ζ_i was significantly lower in the methylated cell type. **B.** Comparison of normalized PRO-seq read depths at differential DNA methylation sites between two cell types. To account for library size and expression levels, raw PRO-seq read counts at each site for each gene were first normalized by dividing them by the total read count for that gene, and then scaled to a range of 0 to 1. CpG sites with a read depth of 0 in both cells were filtered out. As expected, the PRO-seq read depth was significantly higher in the methylated cell type. Asterisks (***) denote t-test p-values < 0.001. **C.** UCSC Genome Browser tracks showing predicted local elongation rate changes at a differentially methylated CpG site (highlighted in the red box). The tracks show lower predicted local elongation rate ζ_i at the methylated CpG site in K562 cells and higher ζ_i at the corresponding non-methylated CpG site in MCF-7 cells.

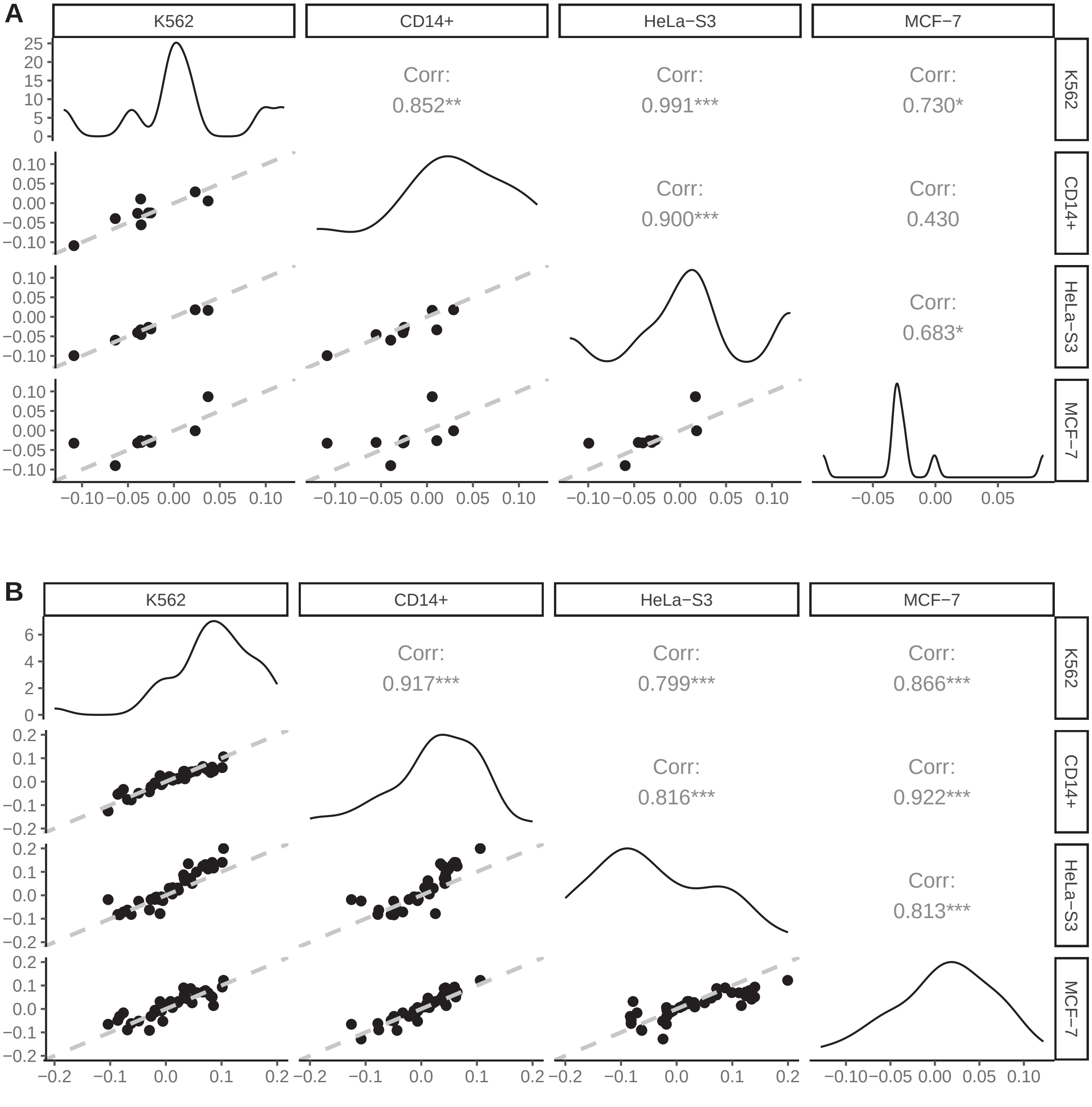

Supplementary Figure S15: **A & B**. The correlation of the estimated κ values across four cell lines, either with the epigenomic features or significant k-mers (N = 45).

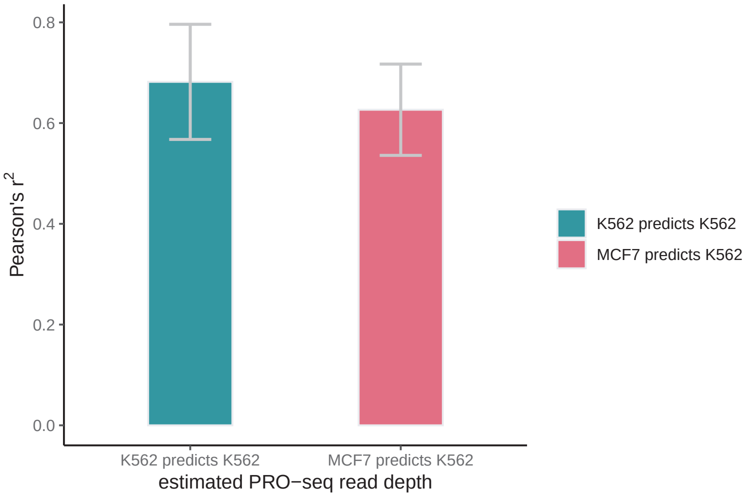

Supplementary Figure S16: Comparison of prediction for K562 cells between models trained on K562 or MCF-7. PRO-seq read depths for K562 cells (averaged in 1kb windows) were predicted using model parameters estimated from MCF-7 data and compared to predictions from a model trained on K562 data. To evaluate variability in prediction accuracy, predictions were repeated 10 times, each using 10 randomly selected, non-overlapping genes. Both models, trained on K562 and MCF-7, demonstrated similar prediction accuracy.

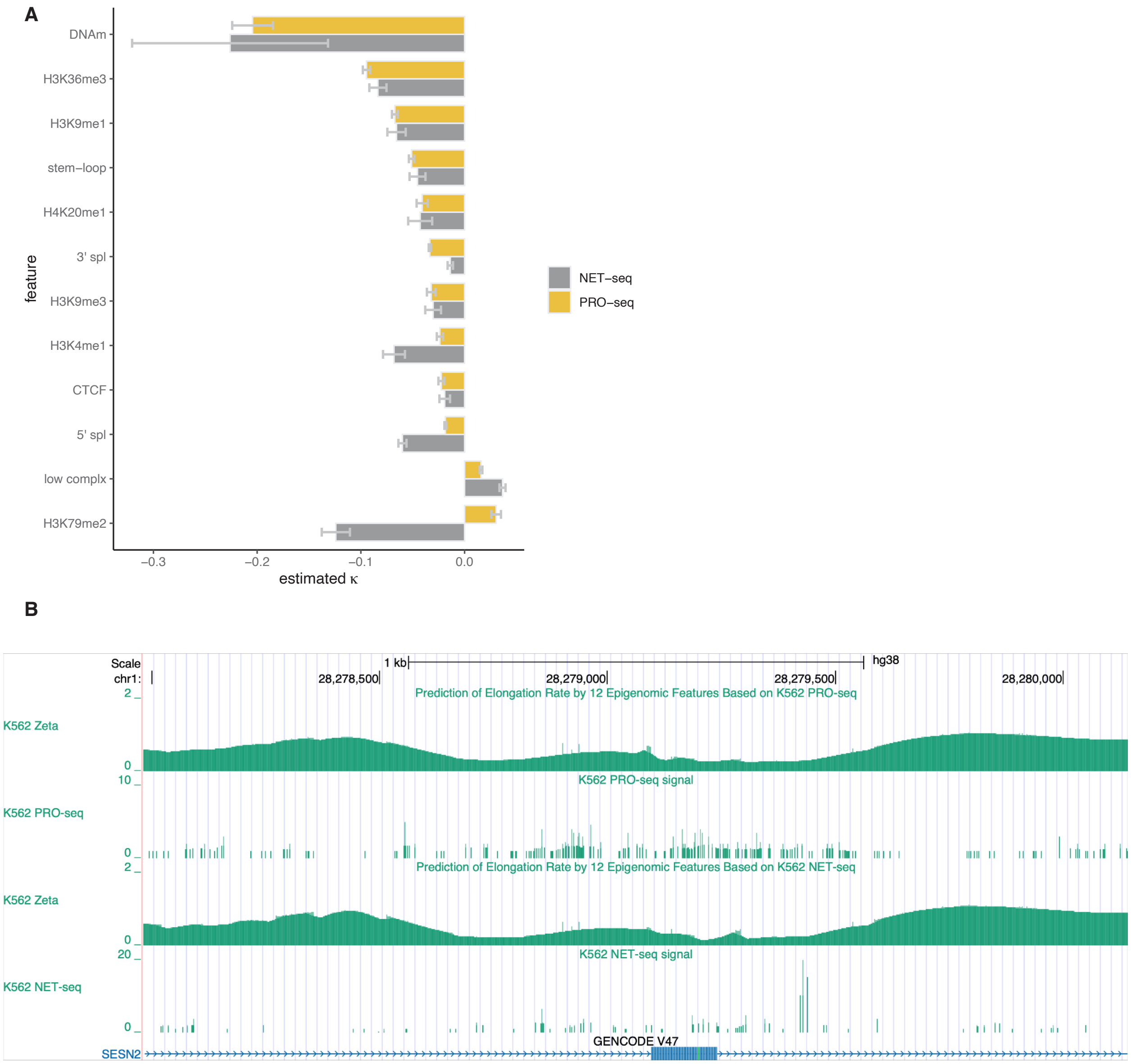

Supplementary Figure S17: Comparison of the K562 epigenomic model using PRO-seq [2] and NET-seq [3]. **A**. Estimated coefficients κ of 12 epigenomic features between the two models. Overall, the estimated κ from NET-seq data aligned closely with those from PRO-seq data, with the exception of H3K79me2. This mark showed a strong negative coefficient with NET-seq but a positive coefficient with PRO-seq. These differences are expected, as PRO-seq captures only transcriptionally active Pol II, whereas NET-seq also detects other Pol II–associated complexes. **B** An example of UCSC Genome Browser tracks showing predicted local elongation rate ζ from both models using PRO-seq and NET-seq, alongside the raw PRO-seq and NET-seq data. Both models similarly predict a reduced local elongation rate around an exon of the *SESN2* gene, as supported by the raw data tracks. However, the NET-seq data appears spikier compared to the PRO-seq data.

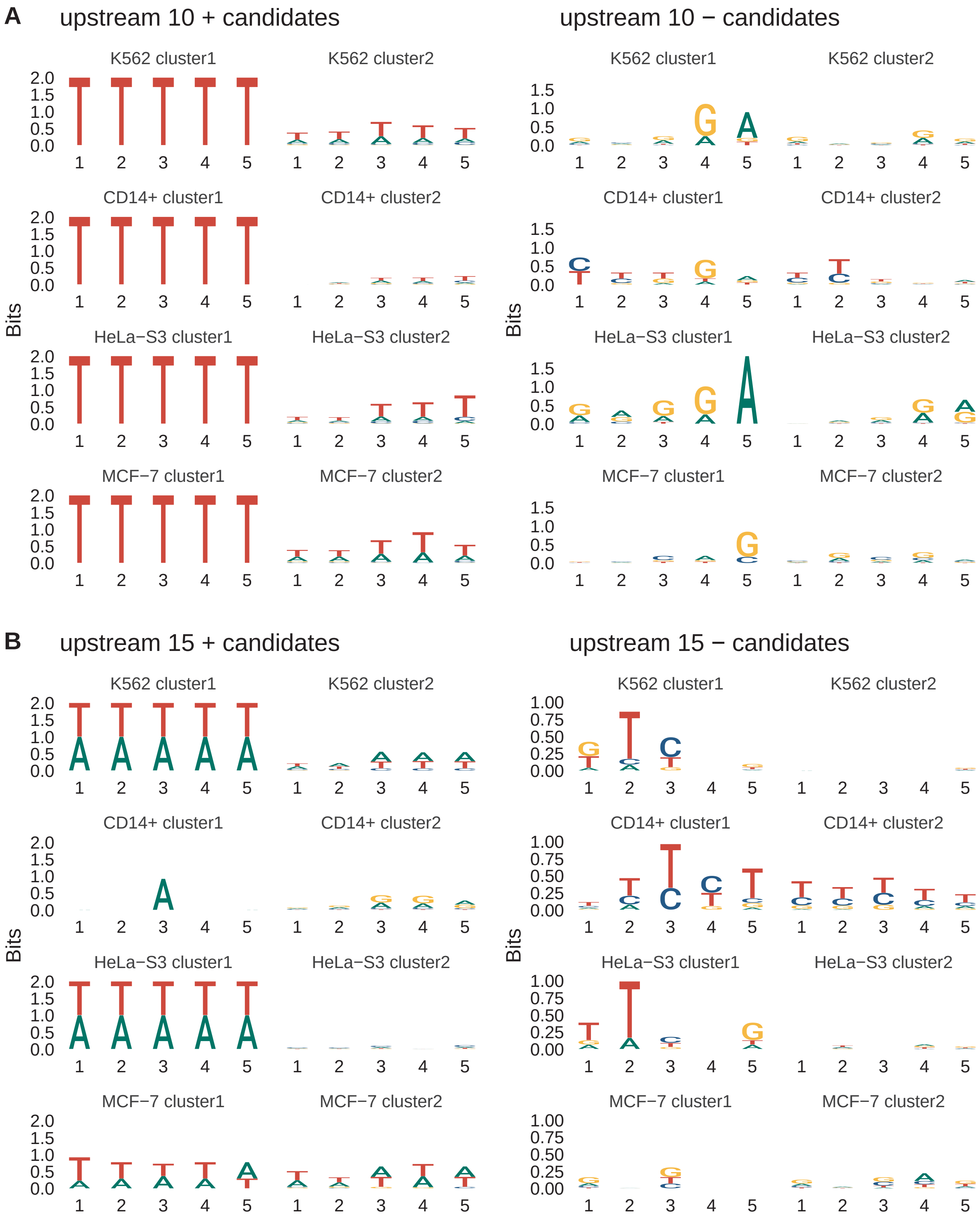

Supplementary Figure S18: **A & B**. Sequence logos summarizing clusters of 5-mers, either ten or fifteen nucleotides upstream of the active site, that are positively (*left*) or negatively (*right*) associated with elongation rate.

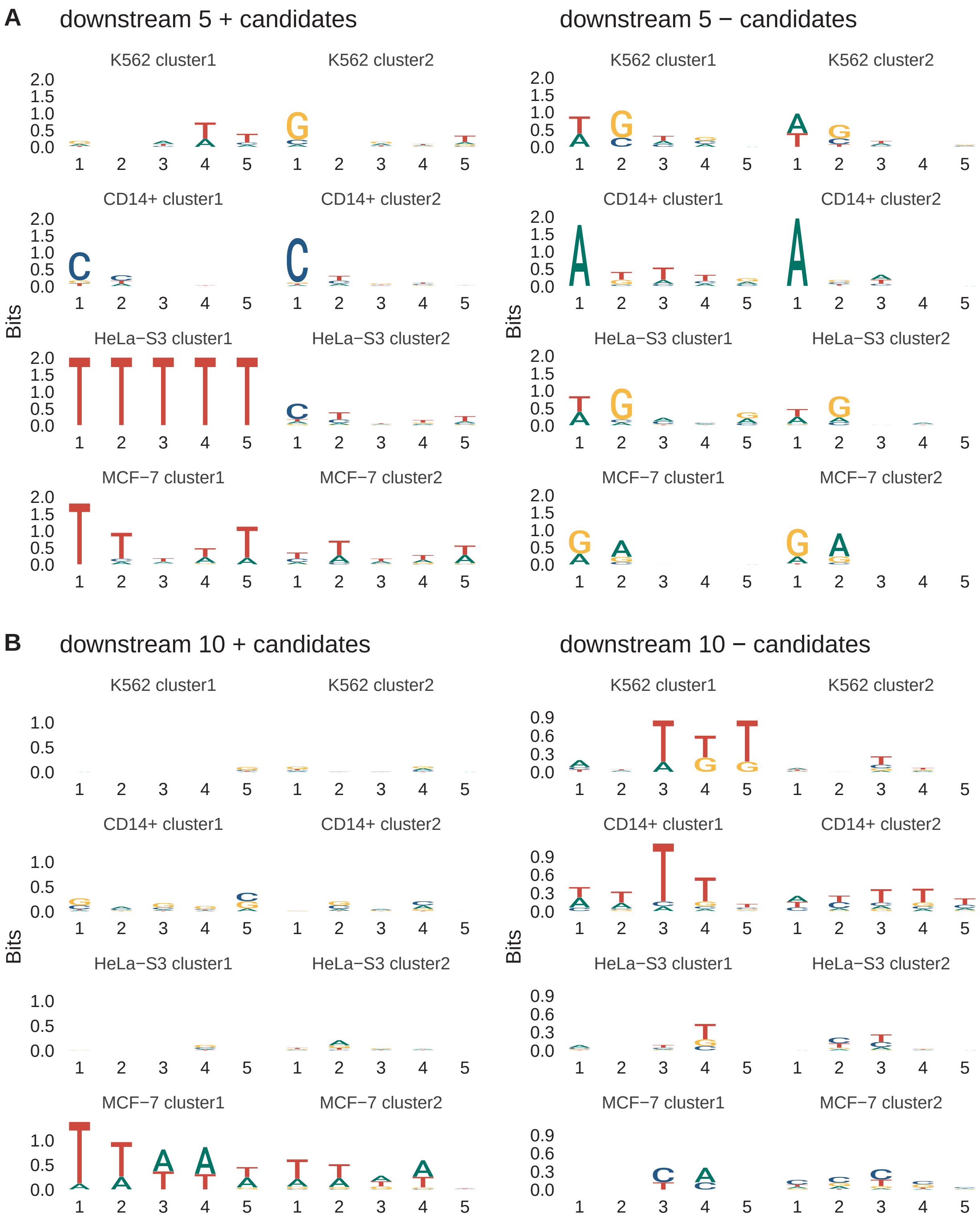

Supplementary Figure S19: **A & B**. Sequence logos summarizing clusters of 5-mers, either five or ten nucleotides downstream of the active site, that are positively (*left*) or negatively (*right*) associated with elongation rate.

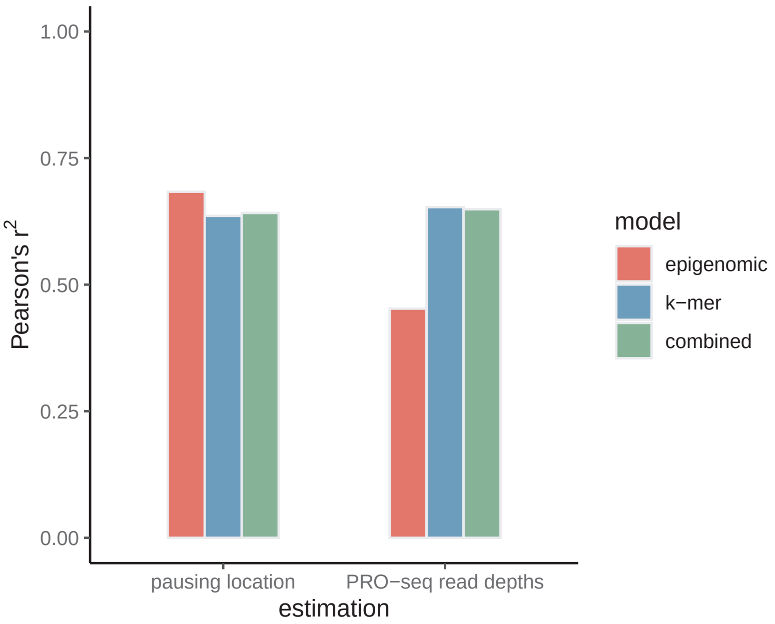

Supplementary Figure S20: The predictive performance of three models, epigenomic, k-mer, and the combined epigenomic and k-mer models, is assessed using held-out data. Prediction accuracy is measured through the estimation of pausing locations and PRO-seq read counts in 1 kbp regions.

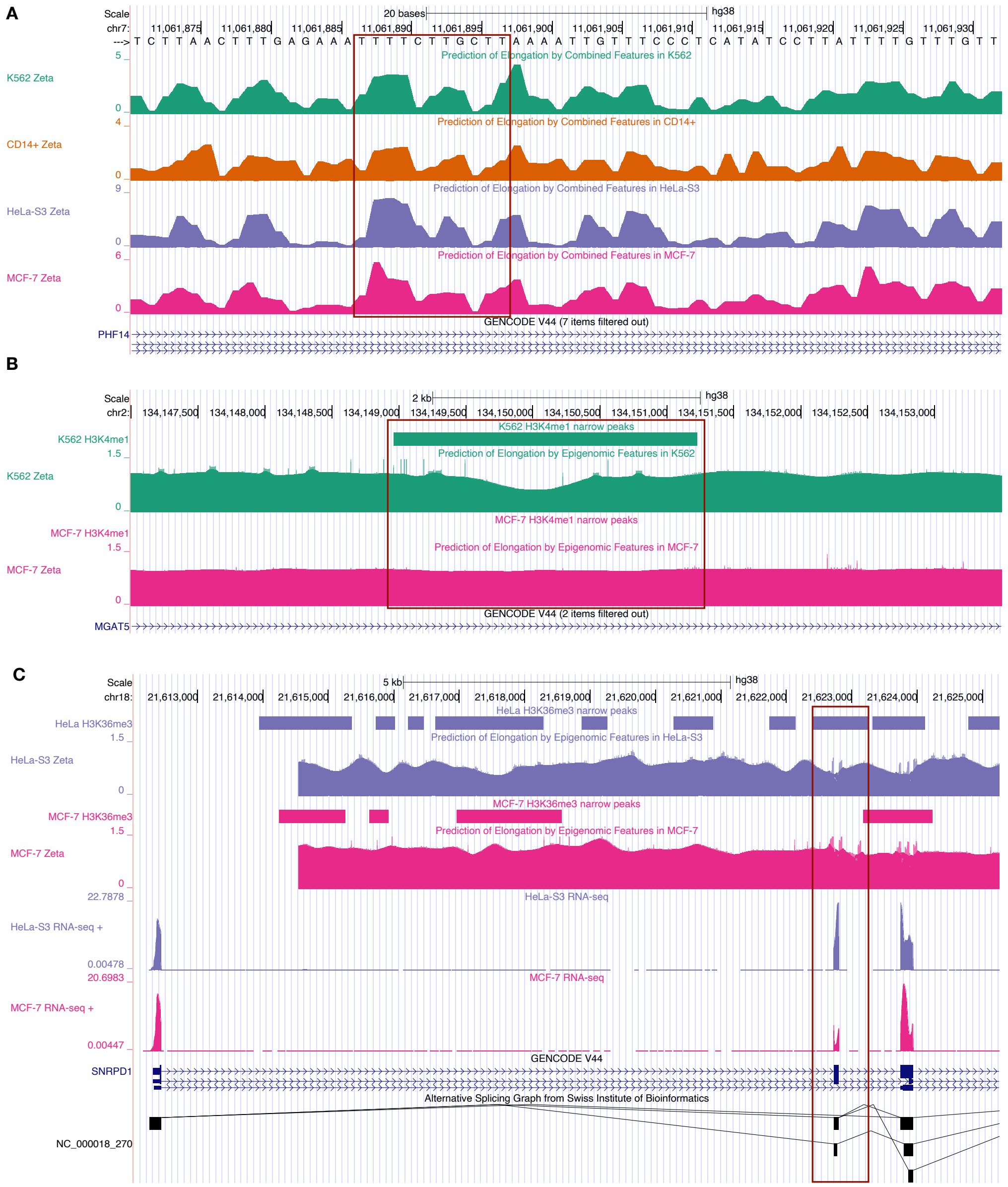

Supplementary Figure S21: Examples of UCSC Genome Browser tracks showing predicted local elongation rates. **A.** Predicted local elongation rates based on the combined k-mer and epigenomic model for the K562, CD14+, HeLa-S3, and MCF-7 cell types in a region of the *PHF14* gene. Elevated predicted rates at poly-T sequences and reductions at cytosines are consistent across cell lines (red boxes). **B.** Predicted local elongation rates based on epigenomic model for K562 and MCF-7 in a region of the *MGAT5* gene. Cell-type-specific reductions in rates associated with H3K4me1 histone marks are highlighted (red boxes). **C.** Predicted local elongation rates based on the epigenomic model for HeLa-S3 and MCF-7 in the alternatively spliced *SNRPD1* gene. H3K36me3 presence correlates with rate reduction, accompanying exon inclusion in HeLa-S3 (red box). Conversely, its absence correlates with no reduction in rates, accompanying exon skipping in MCF-7, as supported by RNA-seq data. In all panels, positive values represent rates on positive strands, while negative values indicate rates on negative strands.

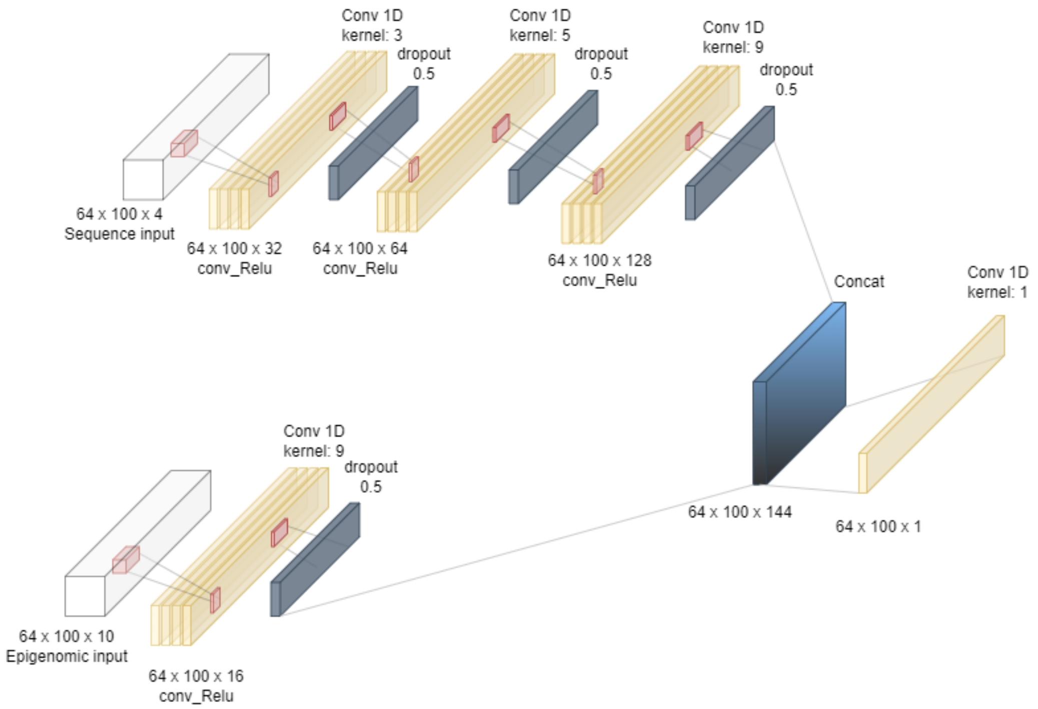

Supplementary Figure S22: Schematic of convolutional neural network (CNN) architecture, generated using draw.io.

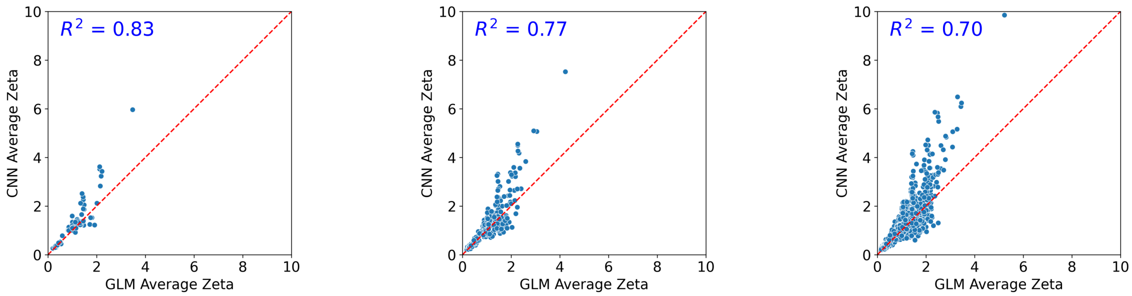

Supplementary Figure S23: Average local elongation rates for k-mers under the GLM and CNN models. Results are shown for all 3-mers (left), 4-mers (center), and 5-mers (right).

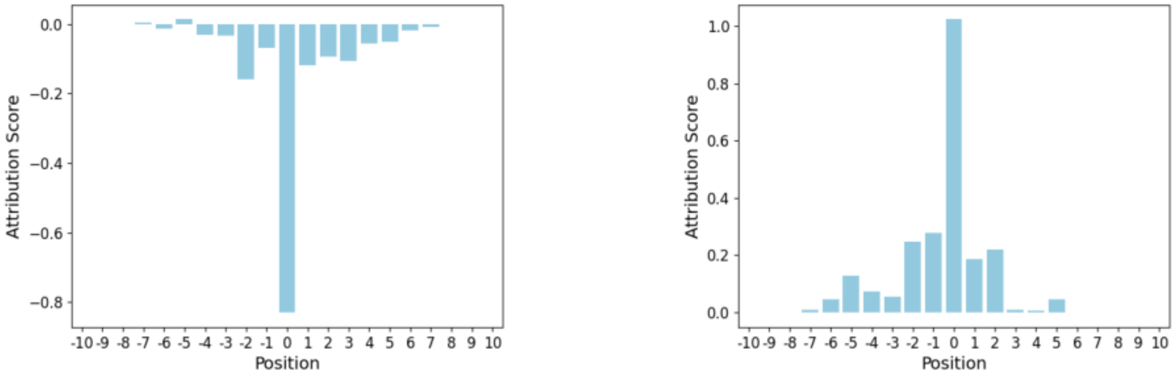

Supplementary Figure S24: Attribution analysis of the CNN. Shown are gradient SHAP scores from the Captum implementation at each nucleotide position relative to the active site. Scores are shown for k-mers associated with a set of low predicted elongation rates (left; based on the $\sim$1500 smallest average $\zeta_{i,j}$ values) and with a set of high predicted elongation rates (right; based on the $\sim$1500 highest average $\zeta_{i,j}$ values). At each position, the average score associated with the reference nucleotide is shown (as opposed to the scores for the three alternative nucleotides).

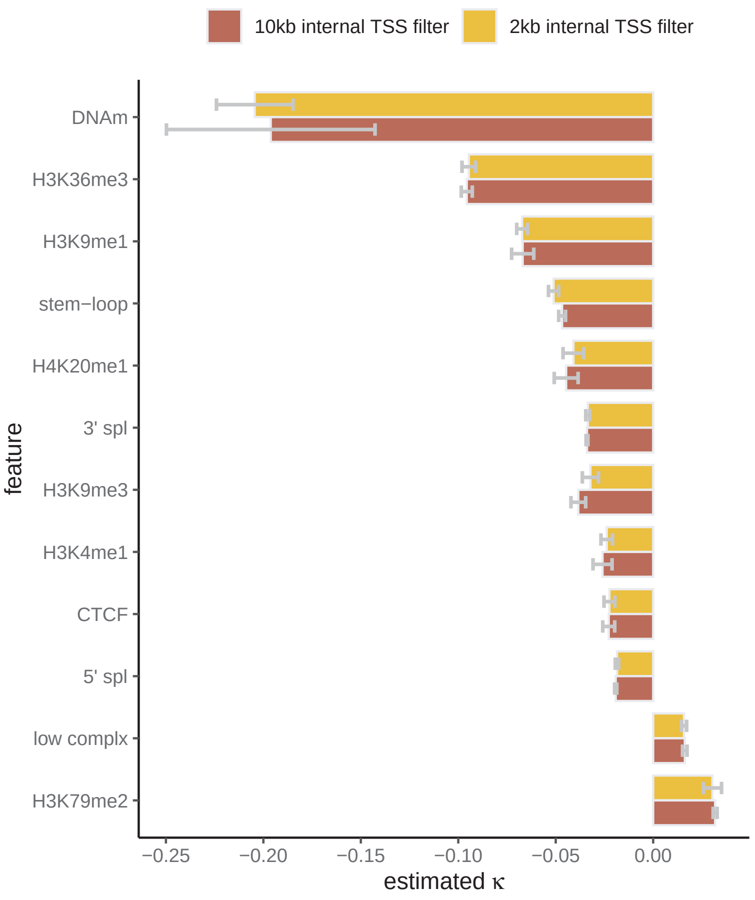

Supplementary Figure S25: Comparison of K562 epigenomic models with 2kb and 10kb internal TSS filters based on GRO-cap signals. The 10kb filter represents a more liberal approach to defining proximity to internal TSS compared to the previously used 2kb filter within gene bodies. The estimated model coefficients show almost no change, with a Pearson's r^2 of 0.997 between the two analyses.

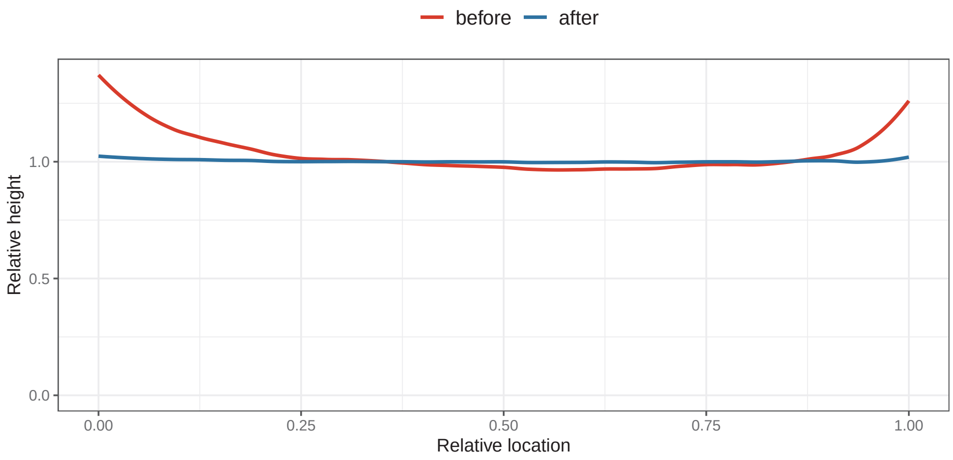

Supplementary Figure S26: A ”U-shape” is evident in the selected gene bodies of K562 PRO-seq data. The red line illustrates the general pattern of the relative height of PRO-seq signals across the relative location of gene bodies. The blue line represents the relative height of PRO-seq signals after correcting the ”U-shape” using the LOESS method (see **Methods**).

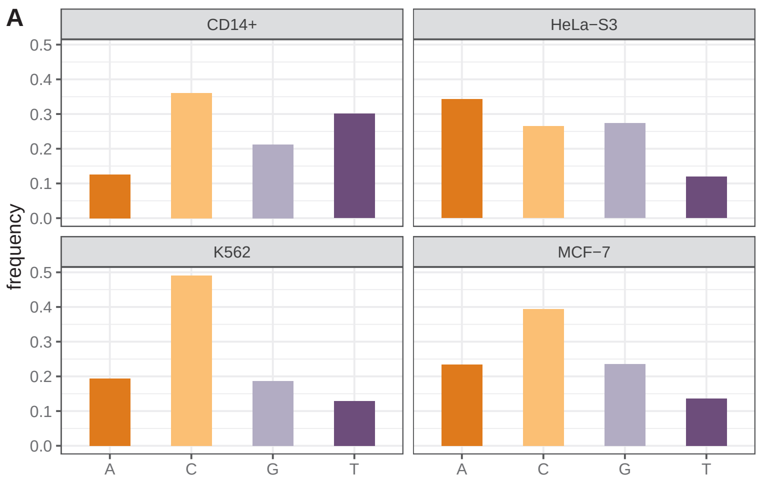

Supplementary Figure S27: The distribution of the $3'$ end base in PRO-seq data across four cell lines.

References

[1] Zhao, Y., Dukler, N., Barshad, G., Toneyan, S., Danko, C. G., and Siepel, A. (2021) Deconvolution of expression for nascent RNA sequencing data (DENR) highlights pre-RNA isoform diversity in human cells. *Bioinformatics,* **37**, 4727–4736.

[2] Dukler, N., Booth, G. T., Huang, Y. F., Tippens, N., Waters, C. T., Danko, C. G., Lis, J. T., and Siepel, A. (2017) Nascent RNA sequencing reveals a dynamic global transcriptional response at genes and enhancers to the natural medicinal compound celastrol. *Genome Res,* **27**, 1816–1829.

[3] Gressel, S., Schwalb, B., and Cramer, P. (2019) The pause-initiation limit restricts transcription activation in human cells. *Nat Commun,* **10**, 3603.
